# Phase Ia/b Multicenter Study of BPM31510IV Targeting Mitochondrial Metabolism/Warburg Effect as Monotherapy and Combination Chemotherapy in Solid Tumor Patients

**DOI:** 10.1158/2767-9764.CRC-25-0507

**Published:** 2025-12-24

**Authors:** Vivek Subbiah, Peter P. Yu, Rangaprasad Sarangarajan, Michael A. Kiebish, Leonardo O. Rodrigues, Gregory M. Miller, Viatcheslav R. Akmaev, Shen Luan, John P. McCook, Nikunj Tanna, Tracey Reilly, Emily Chen, Valerie Bussberg, Shobha Ravipaty, Vivek K. Vishnudas, Stephane Gesta, David Lucius, Can Bruce, Suwagmani Hazarika, Arianne Lyng, Allison Klotz, Vladimir Tolstikov, Janice Stevens, Victoria S. Chua, Elder Granger, Poornima K. Tekumalla, Ely Benaim, Vijay Modur, Marc S. Rudoltz, Paul Y. Song, Scott T. Tagawa, Andrew Hendifer, Sant P. Chawla, Manish A. Shah, David S. Hong, Ralph Zinner, Niven R. Narain, Madappa N. Kundranda

**Affiliations:** 1 MD Anderson Cancer Center, Houston, Texas.; 2 Palo Alto Medical Foundation, Sunnyvale, California.; 3 BPGbio Inc., Waltham, Massachusetts.; 4 Discovery Partners, LLC., Frisco, Texas.; 5 Sarcoma Oncology Center, Santa Monica, California.; 6 MSR Healthcare Consultants, LLC., Pittsfield, Massachusetts.; 7 Weill Cornell Medical College, New York, New York.; 8 Cedars-Sinai Medical Center, Samuel Oschin Cancer Center, Los Angeles, California.; 9 Cancer Center of Southern California, Santa Monica, California.; 10 Department of Dermatology and Cutaneous Surgery, University of Miami Miller School of Medicine, Miami, Florida.; 11 Department of Biochemistry & Molecular Biology, University of Miami Miller School of Medicine, Miami, Florida.; 12 Banner MD Anderson Cancer Center at Banner Gateway Medical Center, Gilbert, Arizona.

## Abstract

**Purpose:**

BPM31510IV, a highly bioavailable intravenously administered coenzyme Q_10_ (CoQ_10_) formulation, was evaluated in a phase Ia/Ib study as monotherapy and in combination with chemotherapy in patients with advanced solid tumors.

**Patients and Methods:**

Using a 3 + 3 design, patients received twice-weekly intravenous infusions of BPM31510IV monotherapy (arm 1) or combined with gemcitabine, 5-fluorouracil/leucovorin, or docetaxel (arm 2); crossover between arms was permitted. Tumor response was assessed by RECIST1.1. Pharmacokinetic and multiomics pharmacodynamic (PD) analyses were performed on plasma and core biopsy samples.

**Results:**

A total of 97 patients were enrolled, 33 in arm 1 and 71 in arm 2 (seven patients crossed from arm 1 to arm 2). The MTD was 171 mg/kg for BPM31510IV monotherapy or with 5-fluorouracil/leucovorin and 110 mg/kg with gemcitabine or docetaxel. Four dose-limiting toxicities occurred (two in monotherapy; two in combination with chemotherapy). Most adverse events were coagulation-related, occurring in 96% of patients (grade ≥3 in 4%). Pharmacokinetics showed dose-proportional increases in CoQ_10_ levels to supraphysiologic concentrations (>200×). In arm 1, there was one (3%) partial response (PR), with stable disease (SD) reported in eight (24%) patients. In arm 2, there was one (1%) PR, with SD reported in 25 (35%) patients. Fluorodeoxyglucose-PET imaging and PD data suggest a change in tumor metabolism from glycolysis to oxidative phosphorylation.

**Conclusions:**

BPM31510IV as monotherapy and in combination with chemotherapy was safe, with preliminary evidence of antitumor activity. High plasma CoQ_10_ levels were achieved, inducing PD responses consistent with mitochondrial metabolic changes. These findings support continued clinical development of BPM31510IV.

**Significance:**

BPM31510IV is a lipid nanodispersion of oxidized CoQ_10_ that alters the Warburg effect and displays anticancer activity. BPM31510IV seems to synergize with chemotherapy to induce cancer cell apoptosis, likely through mitochondrial priming. An initial phase I monotherapy study demonstrated that BPM31510IV is well tolerated in patients with advanced solid tumors. Based on these observations, the safety and preliminary antitumor activity of BPM31510IV were further explored in a phase Ia/Ib study in combination with chemotherapy. Results indicated that BPM31510IV monotherapy and BPM31510IV combined with chemotherapy are well tolerated, demonstrating preliminary evidence of antitumor activity and inducing physiologic and molecular changes consistent with altered mitochondrial metabolism. The most common adverse events were changes in coagulation parameters. Overall, this study provides valuable MTD and surrogate efficacy data that support the continued clinical development of BPM31510IV.

## Introduction

In cancer, metabolic reprogramming supports growth and survival fueled by excessive glucose utilization relative to noncancerous tissue, even in the presence of oxygen (1–3). This phenomenon, known as the Warburg effect, induces a hyperproliferative state and has been demonstrated to play a vital role in the aggressive nature of multiple malignancies, including breast, lung, colorectal, and pancreatic cancers and glioblastoma (2, 4, 5). The development of therapeutic strategies targeting the Warburg effect represents a promising avenue for treating these diseases and improving patient outcomes.

Coenzyme Q10 (CoQ_10_), also known as ubidecarenone, is a lipophilic molecule found in plasma and almost all human cells (6). As an essential component of the mitochondrial electron transport chain, CoQ_10_ also acts as an electron acceptor, regulates permeability of the mitochondrial transition pore, and protects the cellular membrane from oxidative damage (6). Decreased CoQ_10_ levels are associated with aging and numerous conditions, including cardiovascular disease, type 2 diabetes, and cancer (7, 8). Prior studies have suggested that CoQ_10_ may hold potential as an anticancer agent, both alone and by potentiating the activity of other anticancer therapies (8–10). However, clinical testing has been limited by CoQ_10_’s limited solubility in both water and lipids and limited bioavailability when administered orally (11).

**Figure 1.**
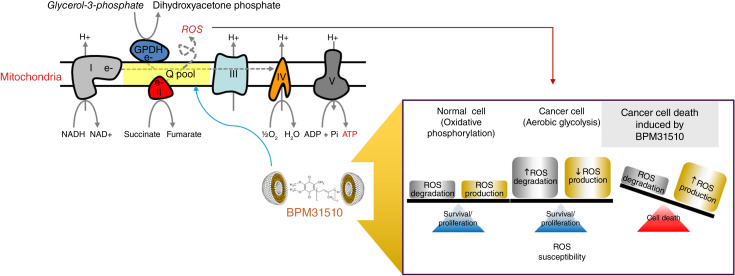
Overview of the proposed mechanism of action for BPM31510IV that includes the induction of ROS production in cancer cells that leads to cell death.

BPM31510IV is a drug–lipid conjugate of oxidized CoQ_10_ in a highly stable nanodispersion formulation for intravenous delivery of supraphysiologic amounts of CoQ_10_ to cells and mitochondria. This formulation improves pharmacologic characteristics of CoQ_10_, increases mitochondrial CoQ_10_ pools, and induces a metabolic switch from glycolysis to oxidative phosphorylation with a concomitant increase in reactive oxygen species (ROS) that results in induction of mitochondrial-mediated apoptosis of cancer cells *in vitro*, as well as in organoids and patient-derived mouse xenografts (12–18). An overview of the proposed mechanism of action for BPM31510IV is shown in Fig. 1. BPM31510IV demonstrated potent anticancer activity associated with elevated levels of mitochondrial CoQ10 in animal models of various cancers, including pancreatic cancer and glioblastoma (17–22), and was well tolerated in a prior phase I clinical study of 50 patients with solid tumors (CTL0510) administered BPM31510IV via 4-hour intravenous infusions three times per week in 28-day cycles. Dose levels ranged from 5.62 to 139 mg/kg, and no dose-limiting toxicities (DLT) were reported (23, 24).

Prior xenograft studies demonstrated that pretreatment with BPM31510IV, followed by chemotherapy with gemcitabine or cytarabine plus doxorubicin, significantly increased median survival in solid tumor and acute leukemia models (25, 26). Observations in pancreatic cancer models also support the concept of “mitochondrial priming,” in which BPM31510IV-mediated induction of mitochondrial oxidative stress synergizes with and enhances chemotherapy-induced apoptosis (25, 27). These findings provide the rationale to evaluate the combination of BPM31510IV plus chemotherapy and to administer BPM31510IV as a single agent for the first 2 weeks to prime the mitochondria prior to initiating chemotherapy. This article describes the results of a Ia/Ib study of BPM31510IV as monotherapy and in combination with chemotherapy to investigate the safety, tolerability, pharmacokinetics (PK), pharmacodynamics (PD), and preliminary evidence of antitumor activity in patients with relapsed and/or refractory solid tumors.

## Patients and Methods

### Clinical trial design

This phase Ia/Ib open-label study used a 3 + 3, nonrandomized, dose-escalation design (28). Eligible patients with metastatic or unresectable solid tumors received two consecutive 48-hour (96 hours total) or 72-hour (144 hours total) intravenous infusions weekly of BPM31510IV (40 mg/mL sterile nanosuspension) as monotherapy (treatment arm 1) or in combination with chemotherapy (treatment arm 2).

During the study, the protocol was amended from 4-day dosing (two 48-hour infusions) for the three lower dose levels to 6-day dosing (two 72-hour infusions) for the three higher dose levels. The protocol was amended again to add additional dosing levels as the MTD had not been reached. The study drug was administered undiluted via a central venous access device, with infusion rate controlled by a programmable ambulatory infusion pump. Dosing was based on patient weight. The starting dose was 66 mg/kg and was escalated by 33% in subsequent cohorts until the MTD was reached. DLTs were to be reported during cycle 1 (duration of 4 weeks for arm 1 and 6 weeks for arm 2). Antitumor activity was assessed after cycle 2 (8 weeks) and every two cycles thereafter for arm 1 and at the end of cycle 1 (6 weeks) and every two cycles thereafter for arm 2. Patients whose disease progressed on BPM31510IV monotherapy in arm 1 were allowed to cross over to arm 2 to receive BPM31510IV plus chemotherapy with Sponsor approval.

The primary objectives were to determine the MTD and assess the safety and tolerability of BPM31510IV monotherapy and in combination with chemotherapy. The secondary objective was to evaluate plasma PK for all patients. Exploratory objectives were to assess (i) antitumor activity of BPM31510IV, (ii) PD correlations of BPM31510IV antitumor activity in plasma, (iii) effects of BPM31510IV on tumor metabolic activity, and (iv) long-term safety and tolerability of repeat BPM31510IV administration as monotherapy and in combination with chemotherapy.

Patients were enrolled from October 2013 to January 2017 at three study sites in the United States; all subjects provided written informed consent. Study protocol and amendments were reviewed and approved by the Institutional Review Board at each center. The trial was conducted in accordance with the International Conference on Harmonization guideline on Good Clinical Practice (E6) and all applicable institutional and local regulatory requirements.

### Eligibility criteria

Patients ≥18 years of age with histologically confirmed metastatic or unresectable solid tumors refractory to standard treatment, an Eastern Cooperative Oncology Group (ECOG) performance status 0 to 2 (29), and adequate bone marrow and organ function, including coagulation parameters [e.g., prothrombin time (PT), international normalized ratio (INR), and activated partial thromboplastin time (aPTT)], were eligible for enrollment. Demographics and representativeness of study participants are listed in Table 1 and Supplementary Table S1, respectively. Detailed inclusion, exclusion, and discontinuation criteria are listed below.

**Table 1.** Demographic information and baseline characteristics for patients enrolled in this phase Ia/Ib study of BPM31510IV.

Characteristic	96-hour infusion		144-hour infusion	
	Arm 1 (*n* = 18)	Arm 2 (*n* = 41)	Arm 1 (*n* = 15)	Arm 2 (*n* = 30)
Age	​	​	​	​
Mean (SD)	59.7 (10.26)	59.6 (10.60)	54.7 (14.85)	54.6 (15.55)
Median	58.5	59.0	56.0	58.5
Range	35, 78	29, 81	29, 77	24, 81
Race	​	​	​	​
Asian	4 (22.2%)	6 (15.4%)	3 (20.0%)	1 (3.3%)
Black/African American	2 (11.1%)	3 (7.7%)	0 (0.0%)	4 (13.3%)
Native Hawaiian/other Pacific Islander	0 (0.0%)	0 (0.0%)	0 (0.0%)	0 (0.0%)
White	12 (66.7%)	30 (76.9%)	12 (80.0%)	25 (83.3%)
Sex	​	​	​	​
Male	10 (55.6%)	22 (53.7%)	8 (53.3%)	15 (50.0%)
Female	8 (44.4%)	19 (46.3%)	7 (46.7%)	15 (50.0%)
Ethnicity	​	​	​	​
Hispanic or Latino	0 (0.0%)	3 (7.5%)	1 (6.7%)	3 (10.0%)
Non-Hispanic or Latino	18 (100.0%)	37 (92.5%)	14 (93.3%)	27 (90.0%)
Previous cancer treatments	​	​	​	​
Chemotherapy	17 (94.4%)	40 (97.6%)	13 (86.7%)	30 (100.0%)
Radiation	5 (27.8%)	17 (41.5%)	10 (66.7%)	15 (50.0%)
Surgery	14 (77.8%)	37 (90.2%)	15 (100.0%)	29 (96.7%)
Number of prior regimens^a^	​	​	​	​
0	1	1	1	0
1	9	4	2	3
2	3	14	0	2
3	1	7	3	5
4	3	6	1	3
5	1	4	0	1
>5 (# of pts, # of prior regimens per patient)	0	4 (6, 7, 10, and 11)	0	2 (6 and 9)
ECOG performance status	​	​	​	​
0	3	5	3	2
1	15	31	12	26
2	—	5	—	2
Primary tumor site	​	​	​	​
Bone sarcoma	0	0	1	3
Breast	1	5	1	1
Central nervous system	0	0	1	0
Colorectal	6	9	4	6
Endometrial	0	1	1	1
Gastro-esophageal	2	4	0	1
Head and neck	1	3	2	2
Hepatobiliary	1	3	2	2
Lung	0	3	2	2
Mesothelioma	0	1	0	0
Neuroendocrine	1	1	0	0
Ovary	1	2	0	1
Pancreatic	1	2	0	1
Prostate	1	1	0	0
Renal cell	1	0	0	1
Salivary gland	0	0	1	1
Soft-tissue sarcoma	1	3	1	2
Testicular	0	0	0	1
Unknown primary	1	1	1	1
Urothelial	0	1	1	1
Uterine sarcoma	0	1	0	1

a Data not available for (a) eight patients in arm 1 who received 144-hour infusion; (b) one patient in arm 2 who received 96-hour infusion; and (c) 16 patients in arm 2 who received 144-hour infusion.

### Inclusion criteria

Patients had to be at least 18 years of age with a histologically confirmed solid tumor that was metastatic or unresectable for which standard measures did not exist or were no longer effective. Patients with primary brain cancer or lymphoma were permitted to be enrolled, and those with brain metastases were allowed if whole-brain radiation was performed and the intracranial tumor was documented to be stable for ≥6 weeks. Additional inclusion criteria included an ECOG performance status ≤2, life expectancy of >3 months, serum electrolytes (i.e., calcium, magnesium, phosphorous, sodium, and potassium) within normal limits (supplementation to maintain normal electrolytes was allowed), adequate coagulation parameters [PT, INR, and PTT ≤1.5 times the upper limit of normal (ULN)], and adequate bone marrow and organ function [absolute neutrophil count ≥1,500 mm^3^, platelets ≥100,000/mm^3^, and hemoglobin ≥9 g/dL; serum creatinine ≤1.8 mg/dL or creatinine clearance >50 mL/minute; and bilirubin ≤1.5 mg/dL and alanine aminotransferase(ALT) and aspartate transaminase (AST) each ≤2.5 times the ULN if there were no liver primary tumors or metastases or ≤5 times the ULN if the liver was involved with tumor]. Sexually active patients and their partners had to agree to use an accepted method of contraception during the study, and female patients of childbearing potential were required to have a negative pregnancy test within 1 week prior to beginning study treatment. All subjects must also have been capable of understanding and complying with the protocol and signing the informed consent document.

### Exclusion criteria and reasons for discontinuation

Exclusion criteria included uncontrolled intercurrent illness (e.g., infection), psychiatric illness/social situations that would limit compliance, use of digitalis alkaloids, colony-stimulating factors, or anticoagulants, and active heart disease, including myocardial infarction within the previous 3 months, symptomatic coronary artery disease, uncontrolled arrhythmias, unstable angina pectoris, or uncontrolled congestive heart failure. Those with uncontrolled or severe bleeding disorders and patients who received chemotherapy or radiotherapy to ≥25% of their bone marrow within 4 weeks of the first dose of study drug, nitrosoureas, or mitomycin C within 6 weeks of the first dose of study drug, or an investigational drug within 30 days of the first dose of study drug, as well as patients whose adverse events (AE) due to investigational drugs or medications administered >4 weeks prior to the first dose of study drug had not recovered to grade ≤1 were also excluded.

Patients were discontinued from the study if they experienced any of the following: (i) documented progressive disease except for patients in arm 1 for whom the treating physician, in conjunction with the Sponsor, considered it in the patient’s best interest to continue BPM31510IV in combination with chemotherapy; (ii) grade ≥2 INR associated with clinically significant bleeding or without bleeding that did not normalize with vitamin K, cryoprecipitate, or fresh frozen plasma within 1 week; (iii) a second asymptomatic grade ≥3 INR; (iv) grade ≥3 aPTT prolongation with or without bleeding or grade ≥2 aPTT prolongation with clinically significant bleeding; (v) treatment delay >2 weeks; (vi) pregnancy; (vii) intercurrent illness that the Investigator deemed would prevent study-related evaluations; and (viii) anaphylaxis. Patients could be withdrawn at the Investigator’s discretion if continuing study therapy was not deemed in the patient’s best interest, and patients could withdraw consent for participation at any time.

### Study cohorts and treatments

Patients in arm 1 received BPM31510IV monotherapy intravenously twice weekly, starting at a dose of 66 mg/kg BPM31510IV per infusion (132 mg/kg/week). BPM31510 was administered using a programmable ambulatory infusion pump. A loading dose (∼8.2% of the total dose) was administered over 1 hour for the first of each two weekly infusions in arm 1, with patients remaining in the infusion suite for at least the first 10 hours of cycle 1 day 1. Cohorts by administered dose are listed in Supplementary Table S2. Patients were monitored for DLTs and AEs requiring hospitalization and discharged if none developed. No more than two dose reductions were permitted, and intrapatient dose escalation was not allowed.

Once single-agent BPM31510IV was deemed safe and tolerable at a specific dose level, arm 2 was opened to enroll patients for treatment with BPM31510IV at a reduced dose level in combination with one of three chemotherapy agents administered once weekly at the following starting doses: gemcitabine (600 mg/m^2^), 5-fluorouracil (5-FU; 350 mg/m^2^) with 100 mg/m^2^ leucovorin (LV), and docetaxel (20 mg/m^2^); allocation to a specific chemotherapy agent was by Investigator decision. Patients were initially administered single-agent BPM31510IV (without a loading dose) for 2 weeks prior to initiation of chemotherapy as preclinical data demonstrated improved efficacy with this regimen. Doses of the chemotherapy agents were increased in subsequent cohorts to their labeled doses, as tolerated (Supplementary Table S3). Administered doses of both BPM31510IV and the chemotherapeutic agent could be escalated simultaneously in cohorts 3 and 4 only if no DLTs were observed in the previous cohorts. Patients in arm 2 who progressed on one chemotherapy agent could not switch to another. If the chemotherapy component was discontinued because of toxicity, patients could continue to receive BPM31510IV monotherapy. Supportive measures, including analgesics, blood transfusions, and antimicrobials, were permitted.

The starting dose of 66 mg/kg BPM31510IV was selected based on safety and PK data from a prior phase I monotherapy study (CTL0510) in which BPM31510IV was administered at doses ranging from 5.62 to 139 mg/kg via 4-hour intravenous infusions three times weekly, with no DLTs observed (23, 24). Given the change to a continuous infusion schedule (48–72 hours) in the current study, the starting dose of 66 mg/kg represented approximately 48% of the maximum safe dose from the prior study, providing an appropriate safety margin in accordance with regulatory guidance for modified administration schedules. The initial chemotherapy doses were deliberately reduced from standard labeled doses to establish the safety profile of combination therapy: gemcitabine at 600 mg/m^2^ represented 60% of the standard 1,000 mg/m^2^ weekly dose; 5-FU/LV at 350/100 mg/m^2^ represented approximately 60% to 88% of standard weekly doses (400–600 mg/m^2^); and docetaxel at 20 mg/m^2^ represented approximately 50% to 67% of standard weekly doses (30–40 mg/m^2^) or 27% of the standard every-3-week dose (75 mg/m^2^). These reduced initial doses, consistent with phase Ib combination study design principles, allowed for systematic dose escalation to labeled doses in subsequent cohorts as safety and tolerability were established (Supplementary Table S3).

Patients whose disease progressed on arm 1 and were considered for crossover to arm 2 were rescreened for eligibility, signed a new informed consent form, and underwent fluorodeoxyglucose (^18^FDG)-PET/CT scans. These patients were only included in the DLT analysis for arm 1; for all other analyses (e.g., PK, PD, and safety), they were included for both arms 1 and 2.

### Infusion protocol and dose-escalation schedule

Administration of BPM31510IV by continuous intravenous infusion using a CADD Prizm VIP (model 6101) pump was based on preclinical data suggesting that longer exposure times and sustained blood levels of BPM31510IV may be correlated with more robust antitumor activity (17, 18). For monotherapy, patients treated with doses ≤110 mg/kg received twice-weekly 48-hour infusions (96-hour total) over four consecutive days (starting on Monday and Wednesday). Those treated with doses ≥137 mg/kg received twice-weekly 72-hour infusions (144-hour total) over six consecutive days (starting on Tuesday and Friday; Supplementary Table S2). When combined with chemotherapy, patients receiving ≤88 mg/kg BPM31510IV were administered consecutive, twice-weekly 48-hour infusions. Patients receiving ≥110 mg/kg were administered consecutive, twice-weekly 72-hour infusions.

A 3 + 3 design (summarized in Supplementary Table S4) was utilized for dose escalation, with decisions on dose and schedule made by the Cohort Review Committee (based on safety, tolerability, and PK data, if available) after the last patient in the dosing cohort completed the first cycle of therapy (4 weeks for arm 1; 6 weeks for arm 2) and met all criteria for DLT assessment.

### AE definition and description

AEs were defined as any unfavorable medical occurrence, including symptoms (e.g., nausea and chest pains), signs (e.g., tachycardia and enlarged liver), or abnormal results (e.g., laboratory findings), appearance of a new disease, or deterioration of a preexisting medical condition. AEs meeting the criteria for “serious” were reported as serious AEs (SAE). AEs occurring on or after initiation of treatment and those present at day 1 of cycle 1 that increased in severity during the study were classified as treatment-emergent AEs (TEAE). Relationship to treatment was assessed by the Investigator as “not related” or “related,” with “related” further qualified as possibly, probably, or definitely. For AEs occurring more than once, only the maximum severity and causality were counted.

### Safety evaluation, DLT, and MTD determination

Safety and tolerability were determined by AEs, hematology, blood chemistry, coagulation parameters, urinalysis, physical examination, interval medical histories, and ECOG performance status. Vital signs, including systolic blood pressure (mm Hg), diastolic blood pressure (mm Hg), pulse rate (beats/minute), oral temperature (°F), and body weight (kg), were assessed on day 1 of each cycle. Electrocardiograms (ECG; 12-lead) were performed at screening and when BPM31510IV exposure was predicted to be at minimum (before dose) and maximum [end of infusion (EOI) on cycle 1 day 1, cycle 1 day 21, and end of treatment].

Toxicities were graded according to the NCI Common Terminology Criteria for Adverse Events (CTCAE) v.4.02 (30).

DLTs were defined as clinically significant AEs or abnormal laboratory values at least possibly related to BPM31510IV during cycle 1. Specific criteria for DLTs were (i) a treatment-related AE that, in the opinion of the Cohort Review Committee, is of potential clinical significance such that further dose escalation would expose patients in higher dose cohorts to risk of irreversible medical harm or require medical treatment to avoid irreversible medical harm; (ii) any grade 3 or 4 nonhematologic toxicity attributed to study drug (with the exception of alopecia or electrolyte abnormalities) including but not limited to grade ≥3 renal and/or liver toxicities and/or grade ≥3 nausea, grade ≥3 diarrhea, and/or grade ≥3 vomiting despite adequate prophylaxis and/or treatment. Grade 3 diarrhea, nausea, and vomiting were considered DLTs unless the toxicity abated to grade ≤2 within 48 hours of instituting maximum supportive therapy; (iii) any Grade ≥4 hematologic toxicity, including but not limited to grade ≥4 anemia or thrombocytopenia or grade ≥4 neutropenia of >5 days duration or of any duration with fever or documented infection; and (iv) any grade 3 coagulation test abnormality (INR/aPTT) with or without bleeding or grade ≥2 prolonged aPTT with clinically significant bleeding. Patients must have received ≥75% of the prescribed dose of BPM31510IV to be eligible for the cohort review assessment in the absence of a DLT.

The MTD was defined as the highest dose at which ≤1 of six patients experienced a DLT during cycle 1, separately for BPM31510IV monotherapy and in combination with each chemotherapy agent, with the MTD criteria for arm 2 including the doses of the chemotherapy agent. If ≥2 patients in a cohort experienced a DLT, the MTD was defined to have been exceeded.

### Response assessments and correlative data

Tumor imaging assessments were performed using CT or MRI at baseline, end of cycle 2, and every two cycles thereafter. The same imaging method was used throughout the study. Tumor response was evaluated using RECIST v.1.1. Change in tumor metabolic activity was assessed using ^18^FDG-PET/CT scans within 2 weeks prior to starting treatment, after 2 weeks of treatment, and at the end of cycle 2. Assessment of tumor imaging was performed centrally (Imaging Endpoints). Five core tumor biopsies were collected at baseline and end of week 2 from patients who consented to participate in exploratory multiomics studies.

### PK analysis

Blood samples for PK analyses were collected from patients undergoing 48-hour BPM31510IV infusion twice weekly [arm 1 (monotherapy) and arm 2 (combination with chemotherapy)] at the following times: cycle 1, week 1 first infusion before dose and 1, 2, 4, 24, and 47.5 hours after the start of the infusion; cycle 1, week 1 second infusion at 1, 2, 4, 24, 44, 46, and 47.5 hours after the start of the second 48-hour dose and 2 and 4 hours after the end of the second 48-hour infusion (approximately 98 and 100 hours after the start of the loading dose; the loading dose was administered during the first hour of the first weekly infusion). For cycle 1, weeks 2, 3, and 4, blood samples were collected before dose and 2, 47.5, and 95.5 hours after the start of the first weekly infusion. For cycles ≥2, samples were collected before dose on days 1 and 15 (first and third weeks) and 95.5 hours after the start of the fourth weekly infusion on day 22.

Blood samples were collected from patients undergoing 72-hour BPM31510IV infusion twice weekly [arm 1 (monotherapy) and arm 2 (combination with chemotherapy)] at the following times: cycle 1, week 1, first infusion before dose and 1, 2, 4, 24, and 71.5 hours after the start of the infusion; second infusion at 1, 2, 68, 70, and 71.5 hours after the start of the second 72-hour dose and 2 and 4 hours after the end of the second 48-hour infusion (approximately 146 and 148 hours after the start of the loading dose). For cycle 1, weeks 2, 3, and 4, blood samples were collected before dose and 2, 71.5, and 143.5 hours after the start of the first weekly infusion. For cycles ≥2, samples were before dose on days 1 and 15 (first and third week) and 143.5 hours after the start of the fourth weekly infusion on day 22.

Plasma BPM31510IV concentrations were determined by LC/MS-MS assays (calibration range, 20–3,000 ng/mL and 3–1,000 μg/mL), with deuterated ubidecarenone-d6 used as an internal standard. For cycle 1, week 1, the following PK parameters were calculated with Phoenix WinNonlin version 8.1 (31) using noncompartmental modeling: (i) C_max_ (peak drug concentration); (ii) T_max_ (time to reach C_max_); (iii) AUC_end1st_ and AUC_end2nd_ [area under the concentration–time curve (AUC) from 0 to 30 minutes before the end of first and second 96- or 144-hour infusion, respectively]; (iv) NC_max_ (C_max_ normalized to weekly dose); (v) NAUC_end1st_ and NAUC_end2nd_ (AUC_end1st_ and AUC_end2nd_ normalized to the weekly dose); (vi) k_el_ (apparent elimination rate constant, determined by regression analysis of the log–linear segment of the plasma concentration–time curve, considered to be reliable if the coefficient of determination, r^2^, is > 0); and (vii) CL (clearance, the amount of drug in the 8-hour collection divided by the AUC from 7.5 to 8 hours after the EOI). Terminal half-life values (t_½_) were calculated as −ln 2/K_el_ based on the plasma concentrations of BPM31510IV at 0.5 hours before and 2 and 4 hours after the EOI for the second weekly infusion; t_1/2_ values were considered reliable if r^2^ > 0.8. Results for AUC_End1st_ and AUC_end2nd_ were assessed for arm 1 and arm 2 to evaluate a possible impact of chemotherapy on PK of BPM31510IV.

### ^18^FDG-PET scanning

Target lesions from ^18^FDG-PET/CT scans were assessed on a time point basis. Metabolically active lesions were identified at baseline and all subsequent PET/CT scans to assess longitudinal changes in ^18^FDG uptake. Regions of interest were posited on each target lesion. The standard uptake values (SUV) such as SUVmax, SUVpeak, and SUVmean, metabolic tumor volume (MTV; the number of voxels with SUVs ≥40% of maximum SUV voxel), and total glycemic index (TGI; SUVmean × MTV) were automatically generated using the mint Lesion Workstation. The sum of SUVmax, SUVpeak, MTV, and TGI of all target lesions per subject was determined and served as a quantitative measure of whole-body metabolic tumor burden.

### Metabolomic, proteomic, and lipidomic (multiomics) analyses (plasma and core biopsies)

Multiomics analyses were performed as described (32). In brief, proteins were extracted from both raw plasma and buffy coat samples. The top 14 most abundant proteins were depleted using the Multi-Affinity Removal Column 14 (Agilent), and protein concentrations were determined using the Bradford Assay. Extracted proteins were reduced, alkylated, and trypsin digested (33), and peptides were labeled using 10-Plex tandem mass tag reagents (Thermo Fisher Scientific). Labeled peptides were then mixed, dried, and desalted on C18 Spin columns and stored at −20°C until LC/MS-MS analysis, using a Waters nanoACQUITY 2D LC system coupled to a Thermo Q Exactive Plus MS. The data were searched against the UniProt database using Proteome Discoverer 1.4 with the SEQUEST and Mascot algorithms.

Plasma samples for metabolomics analysis were prepared as described (31, 34–37). Extracted metabolites were analyzed by (i) gas chromatography with time-of-flight (TOF) high-resolution mass spectrometry (MS) using an Agilent 7890B gas chromatograph fitted with a Gerstel temperature-programmed injector and cooled injection system (CIS 4) and interfaced to a Time-of-Flight Pegasus HT Mass Spectrometer (Leco), (ii) reversed-phase liquid chromatography (LC) with high-resolution MS using the NEXERA XR UPLC system (Shimadzu), coupled with the Triple TOF 6500 System (SCIEX), and (iii) hydrophilic interaction chromatography with LC/MS-MS using the NEXERA XR UPLC system (Shimadzu), coupled with the Triple Quad 5500 System (SCIEX; refs. 31, 34–37). Quality control was performed using metabolite standards mixture and pooled samples, as described (38–41). Raw data were manually inspected, merged, and normalized by the sample median, and metabolite identification was performed by in-house authentic standards analysis using the METLIN, NIST MS, Wiley Registry of Mass Spectral Data, HMDB, MassBank of North America, MassBank Europe, Golm Metabolome Database, SCIEX Accurate Mass Metabolite Spectral Library, MzCloud, and IDEOM databases.

For structural lipidomic analysis, plasma samples were labeled with deuterium-labeled and odd-chain phospholipid standards from diverse lipid classes. Lipid extraction was performed as described, using a customized sequence on a Hamilton Robotics STARlet system (Hamilton). Dried, reconstituted (chloroform:methanol, 1:1, v/v), and diluted lipid extracts were analyzed by electrospray ionization-MS on a TripleTOF 5600+ (SCIEX), coupled to a customized direct-injection loop on an Eksigent microLC200 system (SCIEX; refs. 42, 43). For mediator lipidomic analysis, plasma samples were mixed with deuterium-labeled internal standards and cold methanol and centrifuged at 14,000 × *g* at 4°C for 10 minutes. Supernatants were mixed with acidified water (pH 3.5) prior to loading on C18 SPE columns (Thermo Fisher Scientific, Pierce; ref. 44). Dried and reconstituted (methanol:water, 1:1, v/v) were then centrifuged at 20,000 × *g* at 4°C for 10 minutes and analyzed by LC/MS-MS.

### Multiomics data analysis

Regression analysis was used to identify proteins, lipids, and metabolites significantly altered in response to BPM31510IV treatment. These analyses were performed for each combination of patient, sample type, and trial arm, with each analysis considering two different models for a given dataset. Model 1 is a regression that relates omics features to the fixed-term weeks and hours within the week of cycle 1, and model 2 is limited to the first week of cycle 1 and thus relates omics features to the fixed-term hour. Using this method, many regression models were constructed. To focus only on the most significant molecules for pathway analysis, the most relevant models were selected based on an FDR cutoff of 0.1 for proteins, metabolites, and lipids. For each omics platform, molecules were further filtered based on the presence of a consistent upward or downward trend in the regression models, and only those showing a consistent directionality in >55% of the models were chosen. Features identified from this analysis can have multiple hits if they are found in multiple time points from different patients, in different sample types, or at different time intervals.

For metabolomics analyses, a mixed-effect linear model using the R package lmerTest (45) was fitted to the data for each metabolite using the model formula metabolite ∼ CoQ_10_ + (1|subjID), allowing each patient their own intercept value; metabolite and CoQ_10_ values were log_2_-transformed. FDRs (*q* values) were calculated using the R package qvalue (46). Enrichment analysis for subchemical class sets among the metabolites showing a positive association with CoQ_10_ levels was performed using MetaboAnalyst v.6.0 (https://www.metaboanalyst.ca/). Enrichment of carnitine derivatives (those with names containing the string “YLCARNITINE”) among metabolites positively associated with CoQ_10_, relative to all measured metabolites, was performed with the R package fisher.test.

### Statistical methods

The Safety Population included all patients who received at least one dose of BPM31510IV. PK results were calculated from all patients who had sufficient PK data without significant protocol deviations. The efficacy population comprised all patients who underwent at least one on-treatment tumor imaging assessment or were discontinued prior to the end of the cycle 2 imaging assessment for either PD or toxicity.

All safety analyses were based on the Safety Population. Reporting of AEs was based on the Medical Dictionary for Regulatory Activities and CTCAE v4.02, with data presented by system organ class, preferred term, severity (CTCAE grade), relationship with the study treatment, and dose. The maximum grade for each AE per patient was recorded. Other safety endpoints, including physical examinations, vital signs, clinical laboratory assessments, ECOG status, and ECGs, were summarized descriptively.

Descriptive statistics for continuous data grouped by treatment and dose level were calculated using WinNonlin version 8.1 and included *n* (number of nonmissing observations), mean, standard deviation (SD), median, minimum, and maximum. Statistics for continuous PK data also include geometric mean and coefficient of variation. Arm 1 and arm 2 AUC values were compared using the bioequivalence module of WinNonlin version 8.1. Log-transformed values were used with a parallel design and arm 1 as a reference.

For each metabolite identified from the enrichment analysis, a Fisher exact test (47, 48) was performed, with Benjamini–Hochberg FDR correction (49) to account for multiple comparisons; FDR-corrected *P* values (*q* values) are reported. Additionally, 95% confidence intervals were calculated for selected safety and exploratory variables. Probabilities for progression-free survival (PFS) at each assessment time point were calculated from Kaplan–Meier survival curves.

### Ethical guidelines

This body of work closely followed the ethical guidelines of the Declaration of Helsinki, International Ethical Guidelines for Biomedical Research Involving Human Subjects, Council for International Organizations of Medical Sciences, Belmont Report, or U.S. Common Rule.

## Results

### Patient demographics

**Figure 2.**
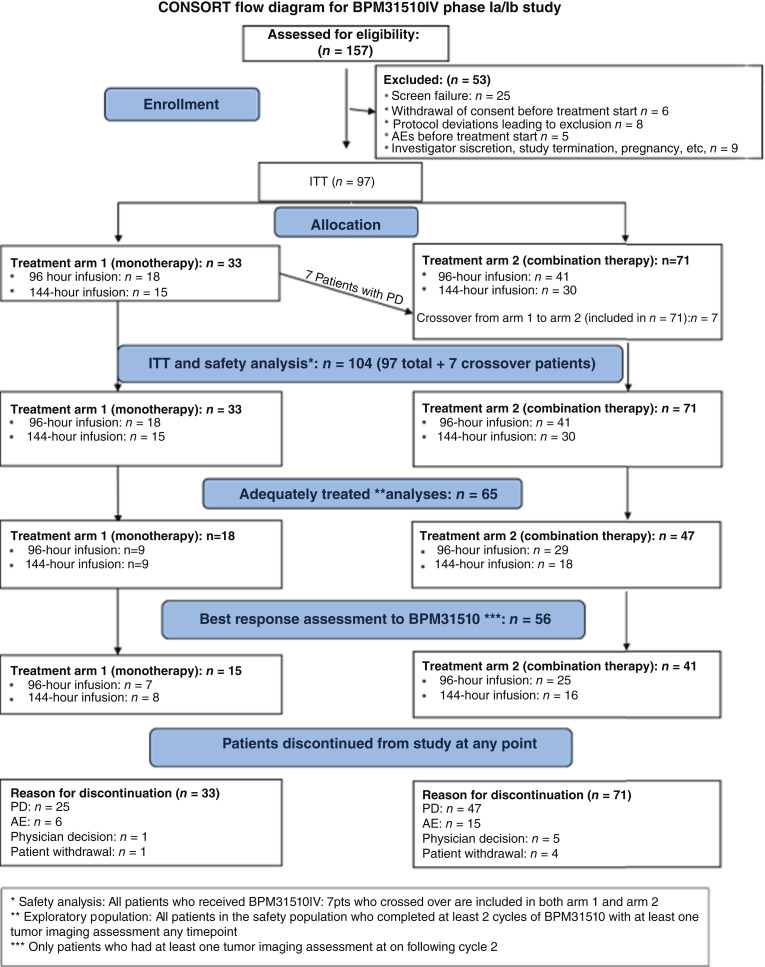
CONSORT diagram of the phase Ia/Ib study evaluating the safety and preliminary antitumor activity of BPM31510IV in patients with solid tumors. ITT, intention to treat.

Among 150 patients assessed for eligibility, 97 patients were enrolled between October 2013 and January 2017 and received at least one dose of BPM31510IV (Fig. 2; Table 1; Supplementary Table S1). Seven patients who progressed on BPM31510IV monotherapy in arm 1 crossed over to arm 2; these patients are included in the counts for both arms below. Thirty-three patients received BPM31510IV monotherapy (arm 1), 18 via 96-hour and 15 via 144-hour infusion; Seventy-one patients received combination therapy (arm 2), 41 via 96-hour and 30 via 144-hour infusion. There were 41 patients in arm 2 treated with the 96-hour infusion who were to receive BPM31510 plus chemotherapy. Of these 41 patients, 14 received gemcitabine, 10 received 5-FU, and 12 received docetaxel. Five patients did not stay on study long enough to receive a first dose of chemotherapy; these patients received BPM31510 for 3, 3, 8, 10, and 15 days, respectively. There were 30 patients in arm 2 who were to receive BPM31510 plus chemotherapy. Of these 30 patients, 11 received gemcitabine, 10 received 5-FU, and five received docetaxel. Four patients did not stay on study long enough to receive a first dose of chemotherapy; these patients received BPM31510 for 7, 11, 15, and 21 days, respectively. Of the seven patients who crossed over from arm 1 to arm 2, one received BPM31510IV + docetaxel, two received BPM31510IV + 5-FU/LV, and four received BPM31510IV + gemcitabine. Eighty-seven patients were included in the PK analysis (*n* = 87), including 25 from arm 1 (96-hour infusion, 13; 144-hour infusion, 12) and 62 from arm 2 (96-hour infusion, 33; 144-hour infusion, 29). All patients (*n* = 97) were included in PD analyses. Tumor imaging was performed on 18 patients from arm 1 (nine patients each from the 96-hour and 144-hour infusion groups) and 47 patients from arm 2 (29 patients from the 96-hour group and 18 patients from the 144-hour infusion group).

### DLTs and determination of MTDs

Four DLTs occurred during the study. Grade 3 elevated ALT, AST, alkaline phosphatase, and γ-glutamyl transferase were reported for a 72-year-old male patient with colon adenocarcinoma who received BPM31510IV monotherapy at 66 mg/kg. Grade 3 prolongation of aPTT was reported in a 77-year-old male patient with appendiceal adenocarcinoma with metastases to the abdominal cavity and a history of prolonged aPTT who received BPM31510IV monotherapy at 171 mg/kg. Grade 3 elevated AST occurred in a 67-year-old female patient with clear cell renal cell carcinoma with metastases to the breast, central nervous system, and pancreas who received BPM31510IV at 137 mg/kg plus gemcitabine (1,000 mg/m^2^), and grade 4 thrombocytopenia was reported in a 28-year-old male patient with a testicular yolk sac tumor and metastases to the lung, lymph nodes, pleura, and thorax who received 137 mg/kg BPM31510IV plus gemcitabine (1,000 mg/m^2^). Both increased AST and thrombocytopenia are known AEs associated with gemcitabine. No DLTs were reported in patients treated with BPM31510 plus either 5-FU/LV or docetaxel.

No MTD was defined for the 96-hour infusion. The following MTDs were defined for the 144-hour infusion of BPM31510IV: (i) 171 mg/kg for BPM31510IV monotherapy, (ii) 110 mg/kg in combination with gemcitabine or docetaxel, and (iii) 171 mg/kg for the combination with 5-FU.

### Patient assessments and AEs

All patients experienced at least one TEAE during the study, most of which were mild or moderate (grade ≤2) in severity. All TEAEs reported at a frequency of ≥10% in any group are listed in Supplementary Tables S5 to S7. The most frequently observed TEAEs (reported in 60%–100% of patients) in both study arms were coagulation-related events. Excluding changes in coagulation parameters, the most common TEAEs of all grades reported in ≥30% of patients were anemia, increased AST, nausea, fatigue, and decreased platelet counts.

Grade ≥3 TEAEs occurred in 22 (67%) patients in arm 1 [96-hour infusion, 11 of 18 (61%); 144-hour infusion, 11 of 15 (73%)] and 47 (66%) patients in arm 2 [96-hour infusion, 23 of 41 (56%); 144-hour infusion, 24/30 (80%); Table 2]. The most common grade ≥3 TEAE in arm 1 was dyspnea in 17% and 13% of patients treated with the 96- and 144-hour infusion, respectively. The most common grade ≥3 TEAE in arm 2 was anemia reported in 20% of patients treated with either the 96- or 144-hour infusion. Other grade ≥3 TEAEs reported in ≥10% of patients include increased AST, ascites, elevated INR, prolonged PT, hypertriglyceridemia, and decreased platelet count.

**Table 2.** Grade ≥3 TEAEs reported in at least two cohorts or in >10% of patients in any cohort (bold).

AEs	96-hour infusion		144-hour infusion	
	Arm 1 (*n* = 18)	Arm 2 (*n* = 41)	Arm 1 (*n* = 15)	Arm 2 (*n* = 30)
Patients with grade ≥3 AEs	11 (61.1%)	23 (56.1%)	11 (73.3%)	24 (80.0%)
Patients with SAEs	8 (44.4%)	22 (53.7%)	10 (66.7%)	19 (63.3%)
Patients with BPM31510IV-related SAEs	1	5	2	3
Blood and lymphatic system	​	​	​	​
Anemia	**2 (11.1%)**	**8 (19.5%)**	1 (6.7%)	**6 (20.0%)**
Neutropenia	—	1 (2.4%)	—	1 (3.3%)
Thrombocytopenia	0 (0.0%)	**5 (12.2%)**	0 (0.0%)	2 (6.6%)
Coagulation	​	​	​	​
Prolonged aPTT	0 (0.0%)	0 (0.0%)	1 (6.7%)	1 (3.3%)
INR, increased	0 (0.0%)	0 (0.0%)	**2 (13.3%)**	0 (0.0%)
Prolonged PT	0 (0.0%)	0 (0.0%)	**2 (13.3%)**	0 (0.0%)
Platelet count decreased	0 (0.0%)	3 (7.3%)	1 (6.7%)	**3 (10.0%)**
Gastrointestinal	​	​	​	​
Abdominal pain	0 (0.0%)	1 (2.4%)	0 (0.0%)	2 (6.7%)
Ascites	0 (0.0%)	1 (2.4%)	**2 (13.3%)**	—
Dysphagia	1 (5.6%)	2 (4.9%)	—	0 (0.0%)
Intestinal obstruction, any	1 (5.6%)	1 (2.4%)	—	—
General/administration site disorders	​	​	​	​
Asthenia	—	2 (4.9%)	1 (6.7%)	1 (3.3%)
Fatigue	0 (0.0%)	1 (2.4%)	1 (6.7%)	2 (6.6%)
Pain	**2 (11.1%)**	0 (0.0%)	—	0 (0.0%)
Infections and infestations	​	​	​	​
Infection	1 (5.6%)	1 (2.4%)	—	—
Sepsis	—	2 (4.9%)	—	1 (3.3%)
Hematology/liver enzymes	​	​	​	​
ALT, increased	1 (5.6%)	2 (4.9%)	1 (6.7%)	1 (3.3%)
AST, increased	1 (5.6%)	**6 (14.6%)**	1 (6.7%)	**4 (13.3%)**
Blood AP, increased	—	1 (2.4%)	0 (0.0%)	1 (3.3%)
Neutrophil count, decreased	0 (0.0%)	1 (2.4%)	—	2 (6.7%)
Metabolism and nutrition	​	​	​	​
Hypertriglyceridemia	1 (5.6%)	1 (2.4%)	**2 (13.3%)**	1 (3.3%)
Musculoskeletal and connective tissue	​	​	​	​
Back pain	**2 (11.1%)**	0 (0.0%)	1 (6.7%)	0 (0.0%)
Psychiatric	​	​	​	​
Anxiety	1 (5.6%)	0 (0.0%)	0 (0.0%)	1 (3.3%)
Respiratory, thoracic, and mediastinal	​	​	​	​
Dyspnea	**3 (16.7%)**	3 (7.3%)	**2 (13.3%)**	**3 (10.0%)**
Hypoxia	1 (5.6%)	1 (2.4%)	—	1 (3.3%)
Pleural effusion	0 (0.0%)	2 (4.9%)	0 (0.0%)	1 (3.3%)
Vascular	​	​	​	​
Embolism	—	1 (2.4%)	1 (6.7%)	1 (3.3%)

Abbreviation: AP, alkaline phosphatase.

A total of 18 (55%) patients in arm 1 and 41 (58%) patients in arm 2 experienced 22 (96-hour infusion, *n* = 11; 144-hour infusion, *n* = 11) and 58 (96-hour infusion, *n* = 32; 144-hour infusion, *n* = 27) SAEs, respectively. Of these, three SAEs in three patients in arm 1 (96-hour infusion, *n* = 1; 144-hour infusion, *n* = 2) and eight SAEs in seven patients in arm 2 (96-hour infusion, *n* = 5; 144-hour infusion, *n* = 3) were attributed as related to the study treatment (Supplementary Table S8). All SAEs considered to be at least possibly attributed to BPM31510IV resolved.

Six (18%) patients from arm 1 and 15 (21%) patients from arm 2 discontinued study drug because of AEs, eight of which were attributed as at least possibly related to BPM31510IV by the Investigator (Supplementary Table S9). There were no deaths on study attributed to BPM31510IV.

### Coagulation parameter abnormalities

Coagulation-associated (CA) TEAEs were the most frequently reported TEAEs (Supplementary Tables S5–S7). Abnormal coagulation parameters were reported in 93 of 97 (96%) patients and were grade ≥3 in four (4%) patients. CA TEAEs stratified by grade, including platelet count/thrombocytopenia, are summarized in Supplementary Table S10. BPM31510IV primarily affected PT/INR and aPTT rather than platelet counts. Most CA TEAEs were grade ≤2, and there were no grade ≥3 CA TEAEs in patients administered BPM31510IV monotherapy in arm 1 via 96-hour infusion. With the 144-hour infusion, there were three patients with grade ≥3 CA TEAEs in arm 1, including prolonged PT and increased INR in each of two (13%) patients and prolonged aPTT and platelet count decreased in one (7%) patient. In arm 2, in which all chemotherapy agents administered with BPM31510IV are myelosuppressive, only one grade ≥3 CA TEAE unrelated to platelet counts was reported, prolonged aPTT in one (3%) patient receiving BPM31510IV via 144-hour infusion.

A total of 17 events associated with bleeding were reported in 16 patients (16%; Supplementary Table S11). Three of these patients were in arm 1 (96-hour infusion, *n* = 1; 144-hour infusion, *n* = 2) and 13 were in arm 2 (96-hour infusion, *n* = 7; 144-hour infusion, *n* = 6); in arm 2, four patients received gemcitabine, seven received 5-FU/LV, and two received docetaxel. Fourteen of the 16 patients reported a total of 45 abnormalities in at least one coagulation parameter, which included aPTT prolongation (*n* = 10), PT prolongation (*n* = 12), increased INR (*n* = 14), decreased platelets (*n* = 4), and thrombocytopenia (*n* = 5). The median time of onset for a bleeding-associated event was 22 days (range, 1–238), and the median duration was 6 days (range, 1–50). Resolution of the AE was reported for nine patients; follow-up was incomplete for the remaining patients.

### Antitumor activity

The best response to BPM31510IV monotherapy (arm 1) was an unconfirmed PR observed at cycle 4 in a 54-year-old male patient with metastatic carcinoma of the soft palate treated at 171 mg/kg (144-hour infusion; Supplementary Table S12). Stable disease (SD) was reported in an additional seven patients, one of whom remained on study into cycle 11. The clinical benefit rate (CBR), defined as a best response of CR, PR, or SD by the end of four cycles, was 11% for the 96-hour infusion and 20% for the 144-hour infusion (Supplementary Fig. S3A and S3B).

The best response when BPM31510IV was combined with chemotherapy was PR. A confirmed PR was reported at the end of cycle 2 in a 59-year-old female with peritoneal mesothelioma treated with BPM31510IV at 66 mg/kg (96 hours) plus gemcitabine at 600 mg/m^2^; this patient remained on study through the end of cycle 6. Unconfirmed responses were reported in two patients after cycle 1, a 64-year-old female with metastatic adenocarcinoma of the pancreas (96-hour infusion) and a 57-year-old female with metastatic breast cancer (144-hour infusion). SD was reported for an additional 26 patients. Six patients remained on study drug beyond four cycles: five cycles (*n* = 1), six cycles (*n* = 2), seven cycles (*n* = 2), and eight cycles (*n* = 1). The CBR was 15% for the 96-hour infusion and 10% for the 144-hour infusion.

The median PFS for patients in arm 1 was 37 and 52 days for patients treated via 96- and 144-hour infusion, respectively, and in arm 2, 65 and 104 days for patients treated via 96- and 144-hour infusion, respectively. There were no relevant differences in PFS between the arms or by the duration of infusion (Supplementary Fig. S1).

**Figure 3.**
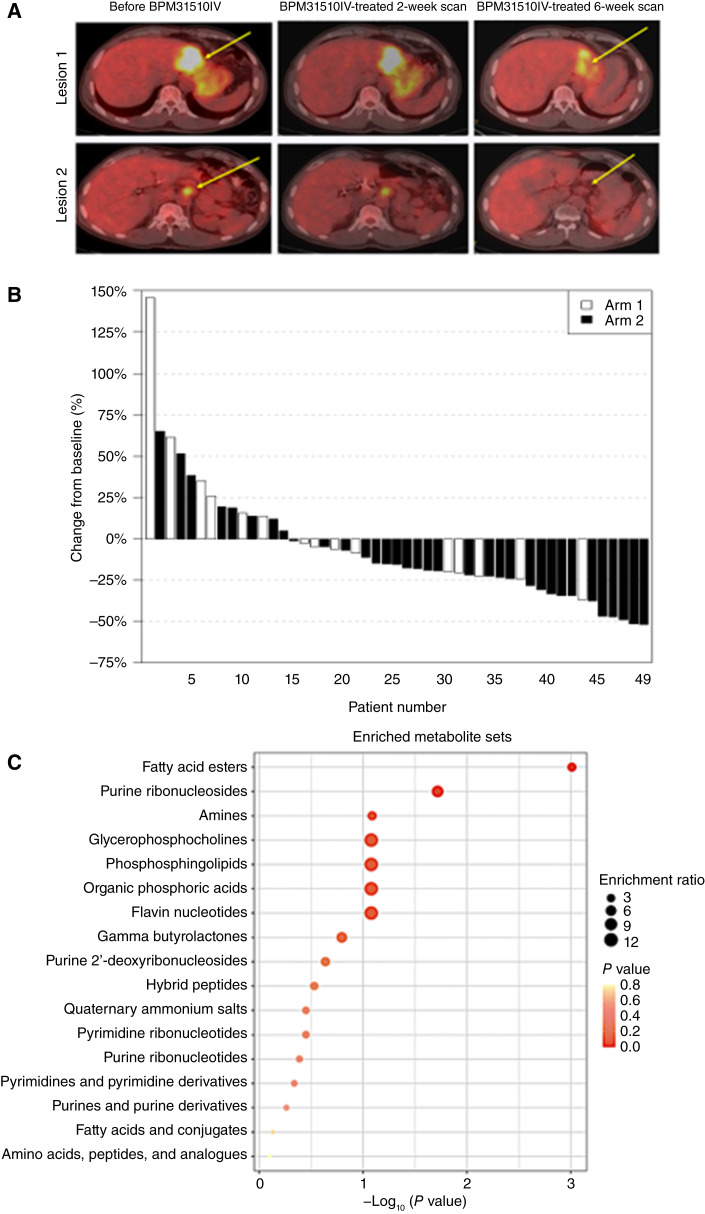
Results from exploratory studies. **A,** Representative ^18^FDG PET/CT scans. A 53-year-old male with gastric adenocarcinoma with liver and lymph node metastases who received BPM31510IV monotherapy at 66 mg/kg. At the end of two cycles of BPM31510IV, there was a 40% decrease in SUV measures, complete resolution of activity in cervical and lymph node metastases, and decreased activity in the liver (yellow arrows). **B,** Waterfall plot showing best change in sum SUVmax for all patients with PET/CT results. For patients who crossed over from arm 1 to arm 2, PET/CT scan data after crossover were omitted from the analysis. **C,** Metabolomics analysis identifies metabolic activity involving fatty acylcarnitines associated with BPM31510IV treatment: enrichment for chemical subclasses among plasma metabolites shows a positive association with plasma CoQ_10_ levels. Chemical subclasses (*y*-axis) are shown in terms of their significance (*x*-axis and dot color) and enrichment ratio relative to the reference metabolome (dot size).

^18^FDG PET/CT imaging was used to qualitatively assess the effect of BPM31510IV on tumor metabolism in 52 patients. Figure 3A shows a representative decrease in SUV in a patient treated with BPM31510IV monotherapy, consistent with a change in tumor metabolism from glycolysis to oxidative phosphorylation. The median overall reduction in ^18^FDG-PET activity based on SUV measures was −4.9%, −11.1%, −19.1%, and −22.6% for BPM31510IV monotherapy and in combination with 5FU/LV, docetaxel, and gemcitabine, respectively; the larger decreases in those treated with combination therapy reflect the antitumor effects of the chemotherapy (Fig. 3B; Supplementary Fig. S4).

### PK

PK parameters for plasma BPM31510IV concentrations at cycle 1 week 1 are summarized in Table 3. Mean plasma concentrations of BPM31510IV during cycle 1 week 1 are shown in Supplementary Fig. S2A–S2D. In general, C_max_, AUC_End1st_, and AUC_end2nd_ increased with increasing BPM31510IV dose. Mean C_max_ values were lower for patients treated via 144-hour versus 96-hour infusion at the same dose level, reflecting a slower infusion rate over a longer period of time in the 144-hour infusion group. In patients treated via 96-hour infusion, C_max_, AUC_End1st_, and AUC_end2nd_ values increased linearly between 50 and 110 mg/kg. In patients treated via 144-hour infusion, AUC_end2nd_ increased proportionally at doses up to 137 mg/kg. At 171 mg/kg, dose-normalized values for C_max_ and AUC in patients treated via 144-hour infusion were similar to dose-normalized values at lower doses, suggesting dose-proportional increases up to 171 mg/kg. Data were insufficient to assess dose proportionality for the 215-mg/kg cohort. Log-transformed dose-normalized AUC values (96- and 144-hour infusions combined) for AUC_End1st_ (arm 1, 4.22; arm 2, 4.13; *P* = 0.435) and AUC_end2nd_ (arm 1, 5.21; arm 2, 5.08 *P* = 0.289) were not significantly different between arms, indicating no relevant impact of the chemotherapy agents on the PK parameters of BPM31510IV. No significant interaction between BPM31510 and the chemotherapy agents was observed. Therefore, PK of the chemotherapy agents was not measured.

**Table 3.** PK parameters from patient serum at cycle 1 week 1.

Dose per infusion (mg/kg)	Infusion time	Arm	*n*	C_max_ (µg/mL)		T_max_ (hour)		AUC_End2nd_ (µg h/mL)	
				Mean	±SD	Mean	±SD	Mean	±SD
50	96 hours	2	9	212	88	79.6	16.0	14,653^a^	6,976^a^
66	96 hours	1	7	346	189	69.8	29.2	29,743^b^	13,678^b^
66	96 hours	2	12	332	201	84.9	18.5	24,350^c^	12,314^c^
88	96 hours	1	2	440	165	84.3	14.6	31,280	9,955
88	96 hours	2	12	406	209	89.7	9.5	30,081^c^	15,051^c^
110	96 hours	1	4	606	206	82.1	23.3	44,770^d^	15,456^d^
110	144 hours	2	17	355	221	113	38	41,985^e^	23,515^e^
137	144 hours	1	3	405	301	119	41	53,505^f^	40,211^f^
137	144 hours	2	8	602	230	126	32	59,667^a^	33,252^a^
171	144 hours	1	7	694	284	122	34	76,579^b^	35,170^b^
171	144 hours	2	3	477	66	143	1	47,744	9,121
215	144 hours	1	2	508	105	141	1	49,269	12,029
215	144 hours	2	1	1,320	—	70.8	—	—	—

Abbreviations: AUC_end2nd_, area under the plasma concentration–time curve from the start of the loading dose to the end of the second infusion; C_max_, maximum concentration; T_max_, time to maximum concentration.

a *n* = 7.

b *n* = 5.

c *n* = 11.

d *n* = 3.

e *n* = 13.

f *n* = 2.

There was no apparent dose or treatment duration dependence observed for mean t_1/2_ values and limited accumulation of BPM31510IV over time. For cycle 1 week 1, the mean estimated t_1/2_ was similar across all dose levels for both treatment arms. Excluding one subject undergoing 96-hour infusion with an estimated t_1/2_ of 15.5 hours, the mean estimated t_1/2_ values for cycle 1 week 1 ranged from 5.39 to 9.63 hours. The mean estimated t_1/2_ for subsequent weeks ranged from 10.0 to 20.8 hours.

### Assessment of proteins and metabolites positively associated with CoQ_10_ upon BPM31510IV treatment

Results from proteomics analysis of plasma, urine, and buffy coat samples revealed distinct signatures associated with the regulation of complement and coagulation events, with prothrombin identified as consistently downregulated (Supplementary Table S13). Additionally, proteomics data show a decrease in vitamin K–dependent protein S and C, suggesting a biochemical rationale for vitamin K supplementation to support maintenance of coagulation parameters.

Assessment of proteins in plasma, urine, and buffy coat samples associated with altered mitochondrial metabolism revealed that key glycolytic enzymes, including enolase, fructose biphosphate aldolase b, and isocitrate dehydrogenase, increased along with critical citric acid cycle enzymes, including transaldolase and malate dehydrogenase (Supplementary Table S14). Plasma and urine metabolite analysis identified an increase in citric acid, the key branch point for citric acid cycle activation (Supplementary Table S15). Lipidomic analysis revealed a consistent increase in plasmalogen phosphocholine molecular species that are enriched in polyunsaturated fatty acids in the SN–2 position and function as potent free-radical scavengers (37) in plasma and urine from BPM31510IV-treated patients (Supplementary Table S16). Together, integrated multiomics analysis assisted in the clarification of the molecular sequelae from BPM31510IV treatment in relation to coagulation events and activation of glycolytic metabolism.

Levels of 327 plasma metabolites were evaluated for their association with CoQ_10_ levels in patients on BPM31510IV monotherapy, using data from all time points and all doses. Among metabolites positively associated with CoQ_10_, 13 of 40 (32.5%) were carnitine derivatives, with names containing the string “YLCARNITINE” (Fig. 3C; Supplementary Table S17). This value represents a 2.4-fold enrichment relative to the ratio for all measured metabolites (*P* = 1.22E−03, by Fisher exact test), suggesting metabolic activity involving fatty acylcarnitines associated with BPM31510IV treatment (Fig. 3C).

## Discussion

The goal of this phase Ia/Ib clinical study was to investigate the safety, PK, and preliminary antitumor activity of BPM31510IV, a nanosuspension formulation of oxidized CoQ_10_ shown to modulate mitochondrial function in cancer cells versus normal cells (17, 18), both as monotherapy and in combination with chemotherapeutic agents. In cancer, the Warburg effect results in mitochondrial overload with production of large amounts of metabolic intermediates to support rapid cell proliferation, potentially representing a vulnerability that can be exploited for effective cancer therapy (2, 50). Thus, an additional goal was to determine whether modulation of mitochondrial activity through supraphysiologic CoQ_10_ supplementation with BPM31510IV could induce PD changes indicative of altered tumor metabolism.

Experiments performed by the Sponsor demonstrated and reports in the literature suggest greater uptake of lipid nanoparticles or liposomal formulations due to differences in the membrane structure and physiology in tumor versus normal cells. Additionally, it is possible that ROS levels in cancer cells are close to a threshold and may be pushed over that threshold via a therapeutic modality, such as the oxidized CoQ10 in BPM31510IV, which raises ROS levels even further and triggers apoptosis. Normal cells are considered protected from undergoing apoptosis because of their lower intrinsic levels of ROS (18).

BPM31510IV administered via a programmable ambulatory infusion pump was shown to be safe and relatively well tolerated, with the most common TEAEs being altered coagulation parameters that can be ameliorated with vitamin K supplementation. Patients treated with higher doses of BPM31510IV via 144-hour infusion as either monotherapy (137–215 mg/kg) or in combination regimens (110–171 mg/kg) seemed to have a higher incidence of coagulation parameter abnormalities than patients receiving lower doses of BPM31510IV via 96-hour infusion as monotherapy (66–110 mg/kg) or in combination regimens (50–88 mg/kg). Other common AEs reported were mostly mild or moderate in severity, including nausea, constipation, fatigue, decreased appetite, and dyspnea, all of which are monitorable and manageable.

Glucose uptake and its anatomic locations in solid tumors can be visualized and localized using ^18^FDG-PET/CT imaging (39). Decreased ^18^FDG uptake following administration of BPM31510IV suggests a switch in metabolism from high-glucose–dependent glycolysis to oxidative phosphorylation. Imaging data showed BPM31510IV dose- and duration-dependent decreases in ^18^FDG-PET activity in a variety of tumors, with greater SUV reductions observed in patients treated with BPM31510IV in combination with standard chemotherapy relative to BPM31510IV alone. The data suggest potential additivity and/or synergy between chemotherapy agents and BPM31510IV, perhaps a greater effect for gemcitabine and docetaxel than 5FU/LV, and support ^18^FDG-PET imaging as a quantitative measure of metabolic response and potential predictor of antitumor activity for future clinical development (51).

To further elucidate the mechanisms underlying the BPM31510IV mechanism of action and uncover molecular profiles associated with its safety and efficacy profile, multiomics analyses were employed to analyze buffy coat and plasma samples. The data reveal distinct molecular signatures associated with coagulation, activation of central carbon metabolism, and generation of plasmalogen lipid molecular species. Critically, insights gained from these comprehensive analyses provide experimental evidence supporting the proposed BPM31510IV mechanism of action (Fig. 1). Multiomics data suggest that alteration of mitochondrial metabolism occurs by increasing key intermediate enzymes in the citric acid cycle, activating lipid metabolism via CoQ_10_ dose-dependent effects on acylcarnitines, and increasing peroxisomal activity and free-radical scavenging, as evidenced by generation of plasmalogen molecular species. The impact of BPM31510 on coagulation parameters seems related to altered regulation of and coagulation events. Proteomics data show this impact as this is likely due to a decrease in proteins dependent on vitamin K, a quinone similar in molecular structure to CoQ10. These findings support the use of vitamin K supplementation to limit these coagulation abnormalities (52).

Proteomics analyses also provide insights into the clotting factors affected by BPM31510IV, with a specific focus on vitamin K metabolism. Natural prenylquinones, such as CoQ_10_, have been shown to inhibit the vitamin K cycle *in vitro* (37). Thus, it was hypothesized that BPM31510IV at supraphysiologic concentrations may affect vitamin K activity on clotting factors. Consistent with this hypothesis, findings from proteomics demonstrate a relationship between vitamin K–dependent protein C and protein S, supporting the use of prophylactic vitamin K supplementation to decrease the risk of bleeding, as noted in laboratory tests by reduced INR and shortened PT and aPTT. In an ongoing study of BPM31510IV in newly diagnosed glioblastoma (NCT04752813), prophylactic vitamin K is being administered to all patients, not only those with grade ≥2 INR as in this study, and seems to have completely mitigated the potential risk of bleeding in these patients (53).

BPM31510IV PK data revealed dose-proportionate increases in exposure to CoQ_10_. Continuous infusion of BPM31510IV maintained plasma levels >100 μg/mL, exposures consistent with BPM31510IV antitumor activity *in vitro*, even at the lowest dose tested (17). This exposure is consistent with omics findings showing effects on mitochondrial metabolism and the most common AEs resulting from a reduction of vitamin K activity. Despite the high percentage of patients with coagulation parameter–related AEs, bleeding events were relatively rare, mostly mild or moderate in intensity, and did not often result in dose modifications of BPM31510IV as monotherapy, BPM31510IV as combination therapy, or the combination chemotherapy agent.

In summary, results from this study show that BPM31510IV, a highly bioavailable form of CoQ_10_, was safe and well tolerated as monotherapy and in combination with cytotoxic chemotherapy, with the caveat that the most common AEs were coagulation abnormalities that seem to be sensitive to vitamin K supplementation. Some antineoplastic activity, PRs, was observed in four patients with heavily pretreated solid tumors. As CoQ_10_ is an integral component of mitochondrial function in all cells, BPM31510IV has the potential to provide clinically relevant antitumor activity for cancers dependent on metabolic hyperactivity, a phenomenon associated with rapid tumor growth (3, 4). These findings support the future clinical development of BPM31510IV, including for the treatment of patients with glioblastoma in an ongoing clinical trial (NCT04752813).

## Supplementary Material

Supplementary Table S1 Representativeness of study participants. Numbers of advanced/Stage IV patients with each cancer type/subtype are also shown.

Supplementary Table S2 BPM31510IV dosing cohorts for monotherapy (Arm 1) and combination therapy (Arm 2).

Supplementary Table S3 Dosing cohorts for BPM31510IV in combination with gemcitabine, 5-fluorouracil plus leucovorin, and docetaxel (Arm 2).

Supplementary Table S4 Dose escalation criteria.

Supplementary Table S5 Treatment-emergent adverse events (TEAEs; all grades) that occurred in ≥10% of patients in any group treated with BPM31510IV.

Supplementary Table S6 Treatment-emergent adverse events (TEAEs; all grades) that occurred in ≥10% of patients in any group treated with BPM31510IV in Arms 1 and 2 stratified by cohort.

Supplementary Table S7 Treatment-emergent adverse events (TEAEs; all grades) that occurred in ≥10% of patients in any group treated with BPM31510IV in Arms 1 and 2 stratified by cohort and chemotherapy received.

Supplementary Table S8 Serious adverse events (SAEs) attributed by the Investigator to the study treatment.

Supplementary Table S9 All treatment-emergent adverse events (TEAEs) resulting in discontinuation of study drug attributed by the Investigator to the study treatment.

Supplementary Table S10 Number of subjects with coagulation-associated treatment-emergent adverse events (TEAEs), including abnormal platelet counts, stratified by grade.

Supplementary Table S11 Adverse events of bleeding reported in study subjects. In total, 17 events were reported in 16 patients, including three patients in Arm 1 who received BPM31510IV monotherapy (96-h infusion, n=1; 144-h infusion, n=2) and 13 in Arm 2 who received BPM31510IV in combination with chemotherapy (96-h infusion, n=7; 144-h infusion, n=6).

Supplementary Table S12 Best response to BPM31510IV patients who had at least one tumor imaging assessment at or following Cycle 2.

Supplementary Table S13 . Proteomic analysis of plasma, urine, and buffy coat revealed a distinct association between BPM31510IV treatment and the regulation of complement and coagulation events. Total hits are the number of significant regression models for each protein (false-discovery rate <0.1). Additional columns refer to the proportion of significant regression models in which the protein is increasing or decreasing; proteins with ten or more hits are listed.

Supplementary Table S14 Proteins detected in plasma, urine, or buffy coat that correlate with metabolic changes. Further information on the column names is provided in the Patients and Methods and the legend for Table S13.

Supplementary Table S15 Metabolites detected in plasma or urine that correlate with systemic protein changes. Further information on the column names is provided in the Patients and Methods and the legend for Table S13.

Supplementary Table S16 Changes in specific plasmalogen molecular species based on regression analysis. Further information on the column names is provided in the Patients and Methods and the legend for Table S13.

Supplementary Table S17 Plasma metabolites positively associated with coenzyme Q10 (CoQ10) levels.

Supplementary Figure S1 Kaplan–Meier curves showing median progression-free survival for patients in each dosing cohort and treatment arm.

Supplementary Figure S2 Mean plasma concentrations of BPM31510IV at Cycle 1 Week 1. Graphs show mean BPM31510IV concentrations for patients receiving 96-h infusion in Arm 1 (A) and Arm 2 (B) and patients receiving 144-h infusion in Arm 1 (C) and Arm 2 (D).

Supplementary Figure S3 Waterfall plots showing best response to BPM31510IV patients who had at least one tumor imaging assessment at or following Cycle 2, by cohort. A., Arm 1. B, Arm 2.

Supplementary Figure S4 Waterfall plot showing different combinations of chemotherapy for ARM2 and different doses of BPM31510IV.

## Data Availability

The datasets used and analyzed in this study were derived from a multiinstitutional, industry-sponsored trial. Data availability is subject to Sponsor discretion and the completion of all clinical trials testing the investigational agent as monotherapy and in combination with chemotherapy. Researchers seeking access to the datasets may submit requests to the Sponsor author, which will be evaluated on a case-by-case basis according to established data-sharing protocols. Additional details about data access procedures and any applicable restrictions are available from the Sponsor upon request.
