# Supplementary material for: Phase Ia/b Multicenter Study of BPM31510IV Targeting Mitochondrial Metabolism/Warburg Effect as Monotherapy and Combination Chemotherapy in Solid Tumor Patients
Source: Cancer Res Commun. 2025 Dec 24;5(12):2207–23. doi: 10.1158/2767-9764.CRC-25-0507 (PMC12727275; doi:10.1158/2767-9764.CRC-25-0507)
Supplement: Supplementary Table S1 — Representativeness of study participants. Numbers of advanced/Stage IV patients with each cancer type/subtype are also shown. [file crc-25-0507_supplementary_table_s1_suppst1.docx]

**Table S1.** Representativeness of study participants. Numbers of advanced/Stage IV patients with each cancer type/subtype are also shown.

| **Characteristic** | **96-h infusion** | | | **144-h infusion** | | |
| --- | --- | --- | --- | --- | --- | --- |
|  | **Arm 1** (*n*=18) | **Arm 2** (*n*=41) | | **Arm 1**  (*n*=15) | **Arm 2** (*n*=30) | |
| Age |  |  | |  |  | |
| Mean (STDEV) | 59.7 (10.26) | 59.6 (10.60) | | 54.7 (14.85) | 54.6 (15.55) | |
| Median | 58.5 | 59.0 | | 56.0 | 58.5 | |
| Range | 35, 78 | 29, 81 | | 29, 77 | 24, 81 | |
| Race |  |  | |  |  | |
| Asian | 4 (22.2%) | 6 (15.4%) | | 3 (20.0%) | 1 (3.3%) | |
| Black/African American | 2 (11.1%) | 3 (7.7%) | | 0 (0.0%) | 4 (13.3%) | |
| Native Hawaiian/Other Pacific Islander | 0 (0.0%) | 0 (0.0%) | | 0 (0.0%) | 0 (0.0%) | |
| White | 12 (66.7%) | 30 (76.9%) | | 12 (80.0%) | 25 (83.3%) | |
| Sex |  |  | |  |  | |
| Male | 10 (55.6%) | 22 (53.7%) | | 8 (53.3%) | 15 (50.0%) | |
| Female | 8 (44.4%) | 19 (46.3%) | | 7 (46.7%) | 15 (50.0%) | |
| Ethnicity |  |  | |  |  | |
| Hispanic or Latino | 0 (0.0%) | 3 (7.5%) | | 1 (6.7%) | 3 (10.0%) | |
| Non-Hispanic or Latino | 18 (100.0%) | 37 (92.5%) | | 14 (93.3%) | 27 (90.0%) | |
| Geography | All patients were enrolled in the USA in the referral sites of the institutions listed in the authors affiliations. | | | | | |
| Overall representativeness of this study | The age distribution of participants in our study is similar to the average age distribution of advanced solid tumor patients in Phase 1 clinical trials.  The male and female populations and race distribution of participants in this study are similar to those in an analysis of a 13,847 patients in 465 protocols. Chihara D, *et al.* Early drug development in solid tumors: Analysis of National Cancer Institute-sponsored phase 1 trials. *The* *Lancet*. 2022 Aug 13;400(10351):512–521. doi: 10.1016/S0140-6736(22)01390-3. PMID: 35964611; PMCID: PMC9477645. | | | | | |
| Primary tumor site |  | |  |  | |  |
| Bone sarcoma | 0 | | 0 | 1 | | 3 |
| Breast | 1 | | 5 | 1 | | 1 |
| Central nervous system | 0 | | 0 | 1 | | 0 |
| Colorectal | 6 | | 9 | 4 | | 6 |
| Endometrial | 0 | | 1 | 1 | | 1 |
| Gastro-esophageal | 2 | | 4 | 0 | | 1 |
| Head and neck | 1 | | 3 | 2 | | 2 |
| Hepatobiliary | 1 | | 3 | 2 | | 2 |
| Lung | 0 | | 3 | 2 | | 2 |
| Mesothelioma | 0 | | 1 | 0 | | 0 |
| Neuroendocrine | 1 | | 1 | 0 | | 0 |
| Ovary | 1 | | 2 | 0 | | 1 |
| Pancreatic | 1 | | 2 | 0 | | 1 |
| Prostate | 1 | | 1 | 0 | | 0 |
| Renal cell | 1 | | 0 | 0 | | 1 |
| Salivary gland | 0 | | 0 | 1 | | 1 |
| Soft tissue sarcoma | 1 | | 3 | 1 | | 2 |
| Testicular | 0 | | 0 | 0 | | 1 |
| Unknown primary | 1 | | 1 | 1 | | 1 |
| Urothelial | 0 | | 1 | 1 | | 1 |
| Uterine sarcoma | 0 | | 1 | 0 | | 1 |

*Data not available for: (a) eight patients in Arm 1 who received 144-h infusion; (b) one patient in Arm 2 who received 96-h; (c) and 16 patients in Arm 2 who received 144-h infusion. ECOG, Eastern Cooperative Oncology Group; STDEV, standard deviation.
