# Supplementary material for: Phase Ia/b Multicenter Study of BPM31510IV Targeting Mitochondrial Metabolism/Warburg Effect as Monotherapy and Combination Chemotherapy in Solid Tumor Patients
Source: Cancer Res Commun. 2025 Dec 24;5(12):2207–23. doi: 10.1158/2767-9764.CRC-25-0507 (PMC12727275; doi:10.1158/2767-9764.CRC-25-0507)
Supplement: Supplementary Table S2 — BPM31510IV dosing cohorts for monotherapy (Arm 1) and combination therapy (Arm 2). [file crc-25-0507_supplementary_table_s2_suppst2.docx]

**Supplementary Table S2.** BPM31510IV dosing cohorts for monotherapy (Arm 1) and combination therapy (Arm 2).

| **Arm 1**  **Cohort*** | **BPM31510IV Dose (2× per week)** | **Dose 1 (Monday)** | | **Dose 2 (Wednesday)** | **Total BPM31510IV Dose per week** |
| --- | --- | --- | --- | --- | --- |
|  |  | **Loading Dose Infused over 1 h (8.2% of dose)** | **Remainder Infused over 47 h** |  |  |
| ‒1 | 50 mg/kg | 4.1 mg/kg | 45.9 mg/kg | 50 mg/kg | 100 mg/kg |
| 1 | 66 mg/kg | 5.4 mg/kg | 60.6 mg/kg | 66 mg/kg | 132 mg/kg |
| 2 | 88 mg/kg | 7.2 mg/kg | 80.8 mg/kg | 88 mg/kg | 176 mg/kg |
| 3 | 110 mg/kg | 9.0 mg/kg | 101.0 mg/kg | 110 mg/kg | 220 mg/kg |
| **Arm 1**  **Cohort** | **BPM31510IV Dose (2× per week)** | **Dose 1 (Tuesday)** | | **Dose 2 (Friday)** | **Total BPM31510IV Dose per week** |
|  |  | **Loading Dose Infused over 1 h (8.2% of dose)** | **Remainder Infused over 71 h** |  |  |
| 4 | 137 mg/kg | 11.2 mg/kg | 125.8 mg/kg | 137 mg/kg | 274 mg/kg |
| 5 | 171 mg/kg | 14.0 mg/kg | 157.0 mg/kg | 171 mg/kg | 342 mg/kg |
| 6 | 215 mg/kg | 17.6 mg/kg | 197.4 mg/kg | 215 mg/kg | 430 mg/kg |
| 7 | 286 mg/kg | 23.4 mg/kg | 262.6 mg/kg | 286 mg/kg | 572 mg/kg |
| 8 | 380 mg/kg | 31.2 mg/kg | 348.8 mg/kg | 380 mg/kg | 760 mg/kg |
| 9 | 505 mg/kg | 41.4 mg/kg | 463.6 mg/kg | 505 mg/kg | 1,010 mg/kg |
| 10 | 672 mg/kg | 55.1 mg/kg | 616.9 mg/kg | 672 mg/kg | 1,344 mg/kg |
| **Arm 2**  **Cohort** | **BPM31510IV Dose (2× per week)** | **Dose 1 (Monday)** | | **Dose 2 (Wednesday)** | **Total BPM31510IV Dose per week** |
|  |  | **Loading Dose Infused over 1 h (8.2% of dose)** | **Remainder Infused over 47 h** |  |  |
| ‒1 | 38 mg/kg | 3.1 mg/kg | 34.9 mg/kg | 38 mg/kg | 76 mg/kg |
| 1 | 50 mg/kg | 4.1 mg/kg | 45.9 mg/kg | 50 mg/kg | 100 mg/kg |
| 2 | 66 mg/kg | 5.4 mg/kg | 60.6 mg/kg | 66 mg/kg | 132 mg/kg |
| 3 | 88 mg/kg | 7.2 mg/kg | 80.8 mg/kg | 88 mg/kg | 176 mg/kg |
| **Arm 2**  **Cohort** | **BPM31510IV Dose (2× per week)** | **Dose 1 (Tuesday)** | | **Dose 2 (Friday)** | **Total BPM31510IV Dose per week** |
|  |  | **Loading Dose Infused over 1 h (8.2% of dose)** | **Remainder Infused over 71 h** |  |  |
| 4 | 110 mg/kg | 9.0 mg/kg | 101.0 mg/kg | 110 mg/kg | 220 mg/kg |
| 5 | 137 mg/kg | 11.2 mg/kg | 125.8 mg/kg | 137 mg/kg | 274 mg/kg |
| 6 | 171 mg/kg | 14.0 mg/kg | 157.0 mg/kg | 171 mg/kg | 342 mg/kg |
| 7 | 215 mg/kg | 17.6 mg/kg | 197.4 mg/kg | 215 mg/kg | 430 mg/kg |
| 8 | 286 mg/kg | 23.4 mg/kg | 262.6 mg/kg | 286 mg/kg | 572 mg/kg |
| 9 | 380 mg/kg | 31.2 mg/kg | 348.8 mg/kg | 380 mg/kg | 760 mg/kg |
| 10 | 505 mg/kg | 41.4 mg/kg | 463.6 mg/kg | 505 mg/kg | 1,010 mg/kg |

*Cohorts ‒1 through 3 received two consecutive 48-h infusions of BPM31510IV, and cohorts 4 through 10 received two consecutive 72-h infusions of BPM31510IV.
