# Supplementary material for: Phase Ia/b Multicenter Study of BPM31510IV Targeting Mitochondrial Metabolism/Warburg Effect as Monotherapy and Combination Chemotherapy in Solid Tumor Patients
Source: Cancer Res Commun. 2025 Dec 24;5(12):2207–23. doi: 10.1158/2767-9764.CRC-25-0507 (PMC12727275; doi:10.1158/2767-9764.CRC-25-0507)
Supplement: Supplementary Table S3 — Dosing cohorts for BPM31510IV in combination with gemcitabine, 5-fluorouracil plus leucovorin, and docetaxel (Arm 2). [file crc-25-0507_supplementary_table_s3_suppst3.docx]

**Supplementary Table S3.** Dosing cohorts for BPM31510IV in combination with gemcitabine, 5-fluorouracil plus leucovorin, and docetaxel (Arm 2).

| **Cohort** | **BPM31510IV Dose** |  | **Gemcitabine Dose**^a^ | **5-Fluorouracil + Leucovorin Doses**b | **Docetaxel Dose** |
| --- | --- | --- | --- | --- | --- |
| ‒1 Gemcitabine | 50 mg/kg^c^ |  | 500 mg/m^2^ | - | - |
| ‒1 BPM31510IV | 38 mg/kg |  | 600 mg/m^2^ | 350 mg/m^2^ + 100 mg/m^2^ | 20 mg/m^2^ |
| 1 | 50 mg/kg |  | 600 mg/m^2^ | 350 mg/m^2^ + 100 mg/m^2^ | 20 mg/m^2^ |
| 2 | 66 mg/kg |  | 600 mg/m^2^ | 350 mg/m^2^ + 100 mg/m^2^ | 20 mg/m^2^ |
| 3 | 88 mg/kg |  | 800 mg/m^2^ | 450 mg/m^2^ + 100 mg/m^2^ | 25 mg/m^2^ |
| 4 | 110 mg/kg |  | 1000 mg/m^2^ | 500 mg/m^2^ + 100 mg/m^2^ | 30 mg/m^2^ |
| 5 | 137 mg/kg |  | 1000 mg/m^2^ | 500 mg/m^2^ +100 mg/m^2^ | 30 mg/m^2^ |
| 6 | 171 mg/kg |  | 1000 mg/m^2^ | 500 mg/m^2^ + 100 mg/m^2^ | 30 mg/m^2^ |
| 7 | 215 mg/kg |  | 1000 mg/m^2^ | 500 mg/m^2^ + 100 mg/m^2^ | 30 mg/m^2^ |
| 8 | 286 mg/kg |  | 1000 mg/m^2^ | 500 mg/m^2^ + 100 mg/m^2^ | 30 mg/m^2^ |
| 9 | 380 mg/kg |  | 1000 mg/m^2^ | 500 mg/m^2^ + 100 mg/m^2^ | 30 mg/m^2^ |
| 10 | 505 mg/kg |  | 1000 mg/m^2^ | 500 mg/m^2^ +100 mg/m^2^ | 30 mg/m^2^ |

^a^Patients receiving 600 mg/m^2^ gemcitabine who experienced a platelet count decrease could have their gemcitabine dose reduced to 500 mg/m^2^ and BPM31510IV continued without a dose reduction.

^b^Leucovorin could be administered prior to 5-fluorouracil, or both drugs could be given concurrently

^c^If the starting dose of 66 mg/kg exceeded the maximum tolerated dose of BPM31510IV, the dose was reduced to 50 mg/kg for Cohort ‒1.
