# Supplementary material for: Phase Ia/b Multicenter Study of BPM31510IV Targeting Mitochondrial Metabolism/Warburg Effect as Monotherapy and Combination Chemotherapy in Solid Tumor Patients
Source: Cancer Res Commun. 2025 Dec 24;5(12):2207–23. doi: 10.1158/2767-9764.CRC-25-0507 (PMC12727275; doi:10.1158/2767-9764.CRC-25-0507)
Supplement: Supplementary Table S4 — Dose escalation criteria. [file crc-25-0507_supplementary_table_s4_suppts4.docx]

**Supplementary Table S4.** Dose escalation criteria.

| **Number of Patients in Cohort with DLT During Cycle 1 (Arms 1 & 2)** | **Dose Escalation Decision Rule** |
| --- | --- |
| 0 out of 3 | Enter 3 patients at the next dose level. |
| 1 out of 3 | Enter 3 more patients at this dose level.  If none of the 3 additional patients has a DLT, proceed to the next level.  If ≥1 of the 3 additional patients has a DLT, stop dose escalation; this dose is declared the MAD. Enter 3 additional patients at the next lowest dose level if only 3 patients were treated previously at that dose. |
| 2 out of 3 | Stop dose escalation; this dose level is declared the MAD (highest dose administered). Enter 3 additional patients at the next lowest dose level if only 3 patients were treated previously at that dose. |
| 1 out of 6 at highest dose level below the maximally administered dose | This is the recommended Phase 2 dose. Enter a total of 12–15 patients at this MTD for Arm 1 monotherapy and 10 patients for each of the 3 chemotherapy subgroups for Arm 2. |

DLT, dose-limiting toxicity; MAD, maximum administered dose; MTD, maximum tolerated dose.
