# Supplementary material for: Phase Ia/b Multicenter Study of BPM31510IV Targeting Mitochondrial Metabolism/Warburg Effect as Monotherapy and Combination Chemotherapy in Solid Tumor Patients
Source: Cancer Res Commun. 2025 Dec 24;5(12):2207–23. doi: 10.1158/2767-9764.CRC-25-0507 (PMC12727275; doi:10.1158/2767-9764.CRC-25-0507)
Supplement: Supplementary Table S5 — Treatment-emergent adverse events (TEAEs; all grades) that occurred in ≥10% of patients in any group treated with BPM31510IV. [file crc-25-0507_supplementary_table_s5_suppst5.docx]

**Supplementary Table S5**. Treatment-emergent adverse events (TEAEs; all grades) that occurred in ≥10% of patients in any group treated with BPM31510IV. TEAEs reported in ≥10% of patients in all four groups are in **bold**.

| **Parameter** | **Arm 1** | | **Arm 2** | |
| --- | --- | --- | --- | --- |
|  | 96-h infusion  *n*=18 | 144-h infusion  *n*=15 | 96-h infusion  *n*=41 | 144-h infusion  *n*=30 |
| Coagulation results |  |  |  |  |
| Prolonged PT | **14 (77.8%)** | **15 (100%)** | **29 (70.7%)** | **18 (60.0%)** |
| INR, increased | **12 (66.7%)** | **13 (86.7%)** | **29 (70.7%)** | **23 (76.7%)** |
| Prolonged aPTT | **11 (61.1%)** | **10 (66.7%)** | **26 (63.4%)** | **22 (73.3%)** |
| Chemistry results |  |  |  |  |
| ALT, increased or decreased | **2 (11.1%)** | **4 (26.7%)** | **8 (19.5%)** | **11 (36.7%)** |
| AST, increased | **4 (22.2%)** | **8 (53.3%)** | **14 (34.1%)** | **19 (63.3%)** |
| AP, increased/abnormal | - | 5 (33.3%) | 5 (12.2%) | 7 (23.3%) |
| Creatinine, increased or decreased | 3 (16.7%) | 1 (6.7%) | 1 (2.4%) | 3 (10.0%) |
| GGT, increased | 1 (5.6%) | 2 (13.3%) | 2 (4.9%) | 2 (6.7%) |
| Blood cholesterol, increased | 1 (5.6%) | 6 (40.0%) | 5 (12.2%) | 7 (23.3%) |
| Blood triglycerides, increased | - | 1 (6.7%) | 1 (2.4%) | 3 (10.0%) |
| Hematology results |  |  |  |  |
| Neutrophil count, decreased | 1 (5.6%) | - | 3 (7.3%) | 3 (10.0%) |
| Platelet count, decreased | **2 (11.1%)** | **4 (26.7%)** | **11 (26.8%)** | **12 (40.0%)** |
| WBC count, decreased | - | - | 3 (7.3%) | 5 (16.7%) |
| Blood and lymphatic system disorders | 7 (38.9%) | 10 (66.7%) | 34 (82.9%) | 25 (83.3%) |
| Anemia | **7 (38.9%)** | **9 (60.0%)** | **31 (75.6%)** | **23 (76.7%)** |
| Thrombocytopenia | 1 (5.6%) | 1 (6.7%) | 12 (29.3%) | 5 (16.7%) |
| Cardiac disorders | 3 (16.7%) | 2 (13.3%) | 6 (14.6%) | 2 (6.7%) |
| Tachycardia | 2 (11.1%) | 2 (13.3%) | 1 (2.4%) | 1 (3.3%) |
| Gastrointestinal disorders | 12 (66.7%) | 13 (86.7%) | 32 (78.0%) | 21 (70.0%) |
| Abdominal pain | 1 (5.6%) | 2 (13.3%) | 1 (2.4%) | 8 (26.7%) |
| Ascites | 1 (5.6%) | 2 (13.3%) | 4 (9.8%) | - |
| Constipation | **4 (22.2%)** | **7 (46.7%)** | **7 (17.1%)** | **6 (20.0%)** |
| Diarrhea | 1 (5.6%) | - | 3 (7.3%) | 3 (10.0%) |
| Dysphagia | 2 (11.1%) | - | 5 (12.2%) | 1 (3.3%) |
| Nausea | **7 (38.9%)** | **8 (53.3%)** | **14 (34.1%)** | **13 (43.3%)** |
| Vomiting | **3 (16.7%)** | **5 (33.3%)** | **11 (26.8%)** | **9 (30.0%)** |
| General disorders | 8 (44.4%) | 12 (80.0%) | 25 (61.0%) | 19 (63.3%) |
| Asthenia | - | 4 (26.7%) | 2 (4.9%) | 2 (6.7%) |
| Fatigue | **6 (33.3%)** | **9 (60.0%)** | **15 (36.6%)** | **10 (33.3%)** |
| Edema, peripheral | 1 (5.6%) | 1 (6.7%) | 7 (17.1%) | 5 (16.7%) |
| Pain | 3 (16.7%) | - | 6 (14.6%) | 2 (6.7%) |
| Pyrexia | 2 (11.1%) | - | 3 (7.3%) | 5 (16.7%) |
| Infections and infestations | 3 (16.7%) | 3 (20.0%) | 16 (39.0%) | 8 (26.7%) |
| Urinary tract infection | - | 1 (6.7%) | 4 (9.8%) | 4 (13.3%) |
| Metabolism and nutrition disorders | 8 (44.4%) | 11 (73.3%) | 28 (68.3%) | 20 (66.7%) |
| Decreased appetite | **3 (16.7%)** | **3 (20.0%)** | **8 (19.5%)** | **6 (20.0%)** |
| Dehydration | 1 (5.6%) | 2 (13.3%) | 4 (9.8%) | 3 (10.0%) |
| Hyperglycemia | - | 2 (13.3%) | 7 (17.1%) | 2 (6.7%) |
| Hyperkalemia | - | - | 1 (2.4%) | 4 (13.3%) |
| Hypertriglyceridemia | **5 (27.8%)** | **5 (33.3%)** | **8 (19.5%)** | **5 (16.7%)** |
| Hypokalemia | - | 1 (6.7%) | 4 (9.8%) | 4 (13.3%) |
| Hypomagnesemia | **4 (22.2%)** | **2 (13.3%)** | **6 (14.6%)** | **5 (16.7%)** |

ALT, alanine aminotransferase; AP, alkaline phosphatase; aPTT, activated partial thromboplastin time; AST, aspartate aminotransferase; GGT, gamma-glutamyl transferase; INR, international normalized ratio; PT, prothrombin time; WBC, white blood cell.

**Table S5 (continued)**. Treatment-emergent adverse events (TEAEs; all grades) that occurred in ≥10% of patients in any group treated with BPM31510IV. TEAEs reported in ≥10% of patients in all four groups are in **bold**.

| **Parameter** | **Arm 1** | | **Arm 2** | |
| --- | --- | --- | --- | --- |
|  | 96-h infusion  *n*=18 | 144-h infusion  *n*=15 | 96-h infusion  *n*=41 | 144-h infusion  *n*=30 |
| Musculoskeletal/connective tissue disorders | 6 (33.3%) | 5 (33.3%) | 10 (24.4%) | 10 (33.3%) |
| Arthralgia | 2 (11.1%) | 3 (20.0%) | 2 (4.9%) | 3 (10.0%) |
| Arthritis | - | 2 (13.3%) | - | - |
| Back pain | 2 (11.1%) | 1 (6.7%) | 3 (7.3%) | 4 (13.3%) |
| Pain in extremity | 2 (11.1%) | 1 (6.7%) | - | 1 (3.3%) |
| Neoplasms benign/malignant/unspecified | - | 2 (13.3%) | 2 (4.9%) | 6 (20.0%) |
| Cancer pain | - | 2 (13.3%) | 1 (2.4%) | 4 (13.3%) |
| Nervous system disorders | 3 (16.7%) | 9 (60.0%) | 7 (17.1%) | 10 (33.3%) |
| Dizziness | - | 2 (13.3%) | 3 (7.3%) | 2 (6.7%) |
| Headache | 2 (11.1%) | 3 (20.0%) | 1 (2.4%) | 4 (13.3%) |
| Neuropathy, peripheral | 1 (5.6%) | 2 (13.3%) | 3 (7.3%) | 1 (3.3%) |
| Psychiatric disorders | 4 (22.2%) | 7 (46.7%) | 7 (17.1%) | 8 (26.7%) |
| Anxiety | 2 (11.1%) | 4 (26.7%) | 4 (9.8%) | 2 (6.7%) |
| Depression | 2 (11.1%) | 1 (6.7%) | - | 4 (13.3%) |
| Insomnia | 2 (11.1%) | 2 (13.3%) | 2 (4.9%) | 4 (13.3%) |
| Renal and urinary disorders | 1 (5.6%) | 3 (20.0%) | 9 (22.0%) | 10 (33.3%) |
| Hematuria | 1 (5.6%) | - | 4 (9.8%) | 4 (13.3%) |
| Proteinuria | - | 2 (13.3%) | 1 (2.4%) | 2 (6.7%) |
| Urinary incontinence | - | - | - | 3 (10.0%) |
| Respiratory, thoracic, mediastinal disorders | 8 (44.4%) | 7 (46.7%) | 19 (46.3%) | 11 (36.7%) |
| Cough | - | 1 (6.7%) | 1 (2.4%) | 4 (13.3%) |
| Dyspnea | **4 (22.2%)** | **4 (26.7%)** | **11 (26.8%)** | **5 (16.7%)** |
| Epistaxis | - | 2 (13.3%) | 1 (2.4%) | 2 (6.7%) |
| Pleural effusion | 3 (16.7%) | 3 (20.0%) | 5 (12.2%) | 2 (6.7%) |
| Skin and subcutaneous tissue disorders | 3 (16.7%) | 3 (20.0%) | 7 (17.1%) | 7 (23.3%) |
| Pruritus | 3 (16.7%) | 1 (6.7%) | 1 (2.4%) | 2 (6.7%) |
| Rash | 1 (5.6%) | 3 (20.0%) | 1 (2.4%) | 2 (6.7%) |
| Vascular disorders | 1 (5.6%) | 3 (20.0%) | 10 (24.4%) | 3 (10.0%) |

ALT, alanine aminotransferase; AP, alkaline phosphatase; aPTT, activated partial thromboplastin time; AST, aspartate aminotransferase; GGT, gamma-glutamyl transferase; INR, international normalized ratio; PT, prothrombin time; WBC, white blood cell.
