# Supplementary material for: Phase Ia/b Multicenter Study of BPM31510IV Targeting Mitochondrial Metabolism/Warburg Effect as Monotherapy and Combination Chemotherapy in Solid Tumor Patients
Source: Cancer Res Commun. 2025 Dec 24;5(12):2207–23. doi: 10.1158/2767-9764.CRC-25-0507 (PMC12727275; doi:10.1158/2767-9764.CRC-25-0507)
Supplement: Supplementary Table S6 — Treatment-emergent adverse events (TEAEs; all grades) that occurred in ≥10% of patients in any group treated with BPM31510IV in Arms 1 and 2 stratified by cohort. [file crc-25-0507_supplementary_table_s6_suppst6.docx]

**Supplementary Table S6.** Treatment-emergent adverse events (TEAEs; all grades) that occurred in ≥10% of patients in any group treated with BPM31510IV in Arms 1 and 2 stratified by cohort.
