# Supplementary material for: Phase Ia/b Multicenter Study of BPM31510IV Targeting Mitochondrial Metabolism/Warburg Effect as Monotherapy and Combination Chemotherapy in Solid Tumor Patients
Source: Cancer Res Commun. 2025 Dec 24;5(12):2207–23. doi: 10.1158/2767-9764.CRC-25-0507 (PMC12727275; doi:10.1158/2767-9764.CRC-25-0507)
Supplement: Supplementary Table S8 — Serious adverse events (SAEs) attributed by the Investigator to the study treatment. [file crc-25-0507_supplementary_table_s8_suppts8.docx]

**Supplementary Table S8**. Serious adverse events (SAEs) attributed by the Investigator to the study treatment.

| **Patient age/sex** | **Arm/**  **Schedule** | **Diagnosis; pertinent history** | **Treatment** | **SAE** | **Action taken** | **Resolved** |
| --- | --- | --- | --- | --- | --- | --- |
| 72-year-old female | Arm 1, 96-h infusion | Esophageal squamous cell carcinoma; history of dysphagia | 110-mg/kg BPM31510IV | Grade 3 increased progressive dysphagia  (possibly related) | Drug withdrawn | Yes |
| 77-year-old male | Arm 1, 144-h infusion | Appendiceal adenocarcinoma with metastases to the abdominal cavity; history of prolonged aPTT | 171-mg/kg BPM31510IV | Grade 3 prolonged aPTT  (definitely related) | Vitamin-K and 2 units fresh frozen plasma; drug withdrawn | Yes |
| 67-year-old male | Arm 1, 144-h infusion | Hepatocellular adenocarcinoma with metastases to the bone | 137-mg/kg BPM31510IV | Grade 3 nausea and vomiting  (possibly related) | Drug interrupted | Yes |
| 70-year-old male | Arm 2, 96-h infusion | Urothelial carcinoma; history of anemia | 66-mg/kg BPM31510IV + 600-mg/m^2^ gemcitabine | (a) Grade 4 anemia;  (b) Grade 4 thrombocytopenia  (both possibly related) | Dose not changed | Yes |
| 64-year-old male | Arm 2, 96-h infusion | Cervical lymph node adenocarcinoma with metastases to the cervical and mediastinal lymph nodes, bone, and left lower lung; history of fatigue | 110-mg/kg BPM31510IV; 50-mg/kg BPM31510IV + 600 mg/m^2^ gemcitabine | Grade 3 weakness/fatigue  (possibly related) | Drug interrupted | Yes |
| 63-year-old female | Arm 2, 96-h infusion | Endometrial adenocarcinoma with metastases to the kidney | 66-mg/kg BPM31510IV + 600-mg/m^2^ gemcitabine | Grade 2 venous thrombosis  (possibly related) | Drug interrupted | Yes |
| 63-year-old female | Arm 2, 96-h infusion | Colon adenocarcinoma; history of constipation | 88-mg/kg BPM31510IV + 450-mg/m^2^ 5‑FU / LV | Grade 2 diarrhea  (possibly related) | Dose not changed | Yes |
| 51-year-old female | Arm 2, 144-h infusion | Lung adenocarcinoma with metastases to the central nervous system | 110-mg/kg BPM31510IV + 1,000-mg/m^2^ gemcitabine | Grade 3 decreased ANC  (possibly related) | Drug interrupted and gemcitabine dose decreased to 800 mg/m^2^ | Yes |
| 54-year-old female | Arm 2, 144-h infusion | Colon adenocarcinoma and metastases to the rectum; history of hepatic atrophy | 137-mg/kg BPM31510IV + 500-mg/m^2^ 5-FU / LV | Grade 3 increased AST (344 U/L)  (possibly related) | Drug withdrawal | Yes |
| 42-year-old male | Arm 2, 144-h infusion | Chromophobe renal cell cancer with metastases to the liver; history of GERD, cancer pain, constipation, and diarrhea | 110-mg/kg BPM31510IV + 1,000-mg/m^2^ gemcitabine | Grade 3 worsening abdominal pain  (possibly related) | Drug withdrawal | Yes |

5-FU, 5-fluorouracil; ANC, absolute neutrophil count; aPTT, activated partial thromboplastin time; AST, aspartate aminotransferase; GERD, gastrointestinal reflux disease; LV, leucovorin.
