# Supplementary material for: Phase Ia/b Multicenter Study of BPM31510IV Targeting Mitochondrial Metabolism/Warburg Effect as Monotherapy and Combination Chemotherapy in Solid Tumor Patients
Source: Cancer Res Commun. 2025 Dec 24;5(12):2207–23. doi: 10.1158/2767-9764.CRC-25-0507 (PMC12727275; doi:10.1158/2767-9764.CRC-25-0507)
Supplement: Supplementary Table S9 — All treatment-emergent adverse events (TEAEs) resulting in discontinuation of study drug attributed by the Investigator to the study treatment. [file crc-25-0507_supplementary_table_s9_suppst9.docx]

**Supplementary Table S9.** All treatment-emergent adverse events (TEAEs) resulting in discontinuation of study drug attributed by the Investigator to the study treatment.

| **Patient age/sex** | **Arm/**  **schedule** | **Diagnosis** | **Treatment** | **SAE** | **Relationship to drug*** |
| --- | --- | --- | --- | --- | --- |
| 72-year-old male | Arm 1, 96-h infusion | Colon adenocarcinoma | 66-mg/kg BPM31510IV | Grade 3 abnormal LFTs (ALT 145 U/L, AST 117 U/L, AP 274 U/L, GGT 373 U/L), considered DLT | Probably |
| 59-year-old female | Arm 1, 144-h infusion | Soft tissue sarcoma on the chest wall with metastases to adrenals, bone liver, lung, and lymph nodes; angiosarcoma and sarcoma metastatic | 137-mg/kg BPM31510IV | Grade 2 ALT increased (202 U/L); Grade 3 AST increased (281 U/L) | Probably |
| 70-year-old male | Arm 2, 96-h infusion | Urothelial carcinoma | 66-mg/kg BPM31510IV + 600-mg/m^2^ gemcitabine | Grade 3 embolism/thrombolytic event | Possibly |
| 64-year-old male | Arm 2, 144-h infusion | Adenocarcinoma in the bladder, lung adenocarcinoma metastatic; history of hypertriglyceridemia and hyperglycemia | 110-mg/kg BPM31510IV + 5-FU / LV | Grade 4 triglyceridemia with triglycerides at 1,334 mg/dL | Probably |
| 67-year-old female | Arm 2, 144-h infusion | Clear cell renal cell carcinoma with metastases to breast, CNS, and pancreas, renal cancer metastatic | 137-mg/kg BPM31510IV + 1,000-mg/m^2^ gemcitabine | Grade 3 AST increased (261 U/L; ALT at 190 U/L), considered a DLT | Possibly |
| 54-year-old female | Arm 2, 144-h infusion | colon adenocarcinoma with metastasis to rectum; history of hepatic atrophy | 137-mg/kg BPM31510IV + 500-mg/m^2^ 5-FU / 500-mg/m^2^ LV | Grade 3 AST increased (344 U/L) | Possibly |
| 28-year-old male | Arm 2, 144-h infusion | Left testicle yolk sac tumor with metastases to lung, lymph nodes, pleura, and thorax; history of anemia | 137-mg/kg BPM31510IV + 1,000-mg/m^2^ gemcitabine | Grade 3/4 thrombocytopenia with a low platelet count of 127×10^9^/L that decreased to 31×10^9^/L, considered a DLT | Definitely |
| 42-year-old male | Arm 2, 144-h infusion | Chromophobe renal cell cancer with metastasis to the liver; history of GERD, cancer pain, constipation, and diarrhea | 110-mg/kg BPM31510IV + 1,000-mg/m^2^ gemcitabine | Grade 3 worsening abdominal pain | Possibly |

5-FU, 5-fluorouracil; ALT, alanine aminotransferase; AP, alkaline phosphatase; AST, aspartate aminotransferase; CNS, central nervous system; DLT, dose-limiting toxicity; GERD, gastrointestinal reflux disease; GGT, gamma-glutamyl transferase; LFTs, liver function tests; LV, leucovorin.
