# Supplementary material for: Phase Ia/b Multicenter Study of BPM31510IV Targeting Mitochondrial Metabolism/Warburg Effect as Monotherapy and Combination Chemotherapy in Solid Tumor Patients
Source: Cancer Res Commun. 2025 Dec 24;5(12):2207–23. doi: 10.1158/2767-9764.CRC-25-0507 (PMC12727275; doi:10.1158/2767-9764.CRC-25-0507)
Supplement: Supplementary Table S10 — Number of subjects with coagulation-associated treatment-emergent adverse events (TEAEs), including abnormal platelet counts, stratified by grade. [file crc-25-0507_supplementary_table_s10_suppst10.docx]

**Supplementary Table S10.** Number of subjects with coagulation-associated treatment-emergent adverse events (TEAEs), including abnormal platelet counts, stratified by grade.

| **Blood/**  **Coagulation TEAEs** | **Arm 1** | | **Arm 2** | |
| --- | --- | --- | --- | --- |
|  | 96-h infusion  *n*=18 | 144-h infusion  *n*=15 | 96-h infusion  *n*=41 | 144-h infusion  *n*=30 |
| Prolonged aPTT | 11 (61.1%) | 10 (66.7%) | 26 (63.4%) | 22 (73.3%) |
| Grade 1 | 8 (44.4%) | 6 (40.0%) | 15 (36.6%) | 13 (43.3%) |
| Grade 2 | 3 (16.7%) | 3 (20.0%) | 11 (26.8%) | 8 (26.7%) |
| Grade 3 | ‒ | 1 (6.7%) | ‒ | 1 (3.3%) |
| INR, increased | 12 (66.7%) | 13 (86.7%) | 29 (70.7%) | 23 (76.7%) |
| Grade 1 | 6 (33.3%) | 5 (33.3%) | 22 (53.7%) | 12 (40.0%) |
| Grade 2 | 6 (33.3%) | 6 (40.0%) | 7 (17.1%) | 11 (36.7%) |
| Grade 3 | ‒ | 2 (13.3%) | ‒ | ‒ |
| Prolonged PT | 14 (77.8%) | 15 (100.0%) | 29 (70.7%) | 18 (60.0%) |
| Grade 1 | 9 (50.0%) | 10 (66.7%) | 26 (63.4%) | 11 (36.7%) |
| Grade 2 | 5 (27.8%) | 3 (20.0%) | 3 (7.3%) | 7 (23.3%) |
| Grade 3 | ‒ | 2 (13.3%) | ‒ | ‒ |
| Platelet count, decr. | 2 (11.1%) | 4 (26.7%) | 11 (26.8%) | 12 (40.0%) |
| Grade 1 | 2 (11.1%) | 2 (13.3%) | 4 (9.8%) | 6 (20.0%) |
| Grade 2 | ‒ | 1 (6.7%) | 4 (9.8%) | 3 (10.0%) |
| Grade 3 | ‒ | 1 (6.7%) | 2 (4.9%) | 2 (6.7%) |
| Grade 4 | ‒ | ‒ | 1 (2.4%) | 1 (3.3%) |
| Thrombocytopenia | 1 (5.6%) | 1 (6.7%) | 12 (29.3%) | 5 (16.7%) |
| Grade 1 | ‒ | ‒ | 7 (17.1%) | 1 (3.3%) |
| Grade 2 | 1 (5.6%) | 1 (6.7%) | ‒ | 2 (6.7%) |
| Grade 3 | ‒ | ‒ | 4 (9.8%) | 1 (3.3%) |
| Grade 4 | ‒ | ‒ | 1 (2.4%) | 1 (3.3%) |

aPTT, activated partial thromboplastin time; INR, international normalized ratio; PT, prothrombin time.
