# Supplementary material for: Phase Ia/b Multicenter Study of BPM31510IV Targeting Mitochondrial Metabolism/Warburg Effect as Monotherapy and Combination Chemotherapy in Solid Tumor Patients
Source: Cancer Res Commun. 2025 Dec 24;5(12):2207–23. doi: 10.1158/2767-9764.CRC-25-0507 (PMC12727275; doi:10.1158/2767-9764.CRC-25-0507)
Supplement: Supplementary Table S11 — Adverse events of bleeding reported in study subjects. In total, 17 events were reported in 16 patients, including three patients in Arm 1 who received BPM31510IV monotherapy (96-h infusion, n=1; 144-h infusion, n=2) and 13 in Arm 2 who received BPM31510IV in combination with chemotherapy (96-h infusion, n=7; 144-h infusion, n=6). [file crc-25-0507_supplementary_table_s11_suppst11.docx]

**Supplementary Table S11.** Adverse events of bleeding reported in study subjects. In total, 17 events were reported in 16 patients, including three patients in Arm 1 who received BPM31510IV monotherapy (96-h infusion, n=1; 144-h infusion, n=2) and 13 in Arm 2 who received BPM31510IV in combination with chemotherapy (96-h infusion, n=7; 144-h infusion, n=6).

| **Adverse event** | **Grade** | | | |
| --- | --- | --- | --- | --- |
|  | **1** | **2** | **3** | **4** |
| Hematuria | 6 | 3 | 1 | 0 |
| Hematochezia | 2 | 0 | 0 | 0 |
| Subconjunctival Hemorrhage | 1 | 0 | 0 | 0 |
| Vaginal Bleeding | 1 | 0 | 0 | 0 |
| Post-menopausal bleeding | 1 | 0 | 0 | 0 |
| GI bleeding | 0 | 0 | 1 | 0 |
| Rectal Bleeding | 1 | 0 | 0 | 0 |
