# Supplementary material for: Phase Ia/b Multicenter Study of BPM31510IV Targeting Mitochondrial Metabolism/Warburg Effect as Monotherapy and Combination Chemotherapy in Solid Tumor Patients
Source: Cancer Res Commun. 2025 Dec 24;5(12):2207–23. doi: 10.1158/2767-9764.CRC-25-0507 (PMC12727275; doi:10.1158/2767-9764.CRC-25-0507)
Supplement: Supplementary Table S12 — Best response to BPM31510IV patients who had at least one tumor imaging assessment at or following Cycle 2. [file crc-25-0507_supplementary_table_s12_suppst12.docx]

**Supplementary Table S12.** Best response to BPM31510IV patients who had at least one tumor imaging assessment at or following Cycle 2.

| **Response** | **Arm 1, 96-h infusion** | | | |
| --- | --- | --- | --- | --- |
|  | Cohort 1 (66 mg/kg) | Cohort 2 (88 mg/kg) | Cohort 3 (110 mg/kg) | Overall |
| *n* | 4 | 3 | 0 | 7 |
| CR | 0 (0%) | 0 (0%) | 0 (0%) | 0 (0%) |
| PR | 0 (0%) | 0 (0%) | 0 (0%) | 0 (0%) |
| SD | 3 (75%) | 2 (67%) | 0 (0%) | 5 (71.4%) |
| PD | 1 (25%) | 0 (0%) | 0 (0%) | 1 (14.3%) |
| NE | 0 (0%) | 1 (33%) | 0 (0%) | 1 (14.3%) |
|  | **Arm 1, 144-h infusion** | | | |
|  | Cohort 4 (137 mg/kg) | Cohort 5 (171 mg/kg) | Cohort 6 (215 mg/kg) | Overall |
| *n* | 3 | 3 | 2 | 8 |
| CR | 0 (0%) | 0 (0%) | 0 (0%) | 0 (0%) |
| PR | 0 (0%) | 1 (33%) | 0 (0%) | 1 (12.5%) |
| SD | 2 (67%) | 0 (0%) | 0 (0%) | 2 (25%) |
| PD | 1 (33%) | 2 (67%) | 2 (100%) | 5 (62.5%) |
| NE | 0 (0%) | 0 (0%) | 0 (0%) | 0 (0%) |
|  | **Arm 2, 96-h infusion** | | | |
|  | Cohort 1 (50 mg/kg) | Cohort 2 (66 mg/kg) | Cohort 3 (88 mg/kg) | Overall |
| *n* | 9 | 8 | 8 | 25 |
| CR | 0 (0%) | 0 (0%) | 0 (0%) | 0 (0%) |
| PR | 0 (0%) | 1 (12.5%) | 0 (0%) | 1 (4%) |
| SD | 5 (56%) | 4 (50%) | 5 (62.5%) | 14 (56%) |
| PD | 3 (33%) | 1 (12.5%) | 1 (12.5%) | 5 (20%) |
| NE | 1 (11%) | 2 (25%) | 2 (25%) | 5 (20%) |
|  | **Arm 2, 144-h infusion** | | | |
|  | Cohort 4 (110 mg/kg) | Cohort 5 (137 mg/kg) | Cohort 6 (171 mg/kg) | Overall |
| *n* | 9 | 4 | 3 | 16 |
| CR | 0 (0%) | 0 (0%) | 0 (0%) | 0 (0%) |
| PR | 0 (0%) | 0 (0%) | 0 (0%) | 0 (0%) |
| SD | 6 (67%) | 3 (75%) | 3 (100%) | 12 (75%) |
| PD | 3 (33%) | 1 (25%) | 0 (0%) | 4 (25%) |
| NE | 0 (0%) | 0 (0%) | 0 (0%) | 0 (0.0%) |

CR, complete response; PR, partial response; SD, stable disease; PD, progressive disease; NE, non-evaluable.
