# Supplementary material for: Phase Ia/b Multicenter Study of BPM31510IV Targeting Mitochondrial Metabolism/Warburg Effect as Monotherapy and Combination Chemotherapy in Solid Tumor Patients
Source: Cancer Res Commun. 2025 Dec 24;5(12):2207–23. doi: 10.1158/2767-9764.CRC-25-0507 (PMC12727275; doi:10.1158/2767-9764.CRC-25-0507)
Supplement: Supplementary Table S13 — . Proteomic analysis of plasma, urine, and buffy coat revealed a distinct association between BPM31510IV treatment and the regulation of complement and coagulation events. Total hits are the number of significant regression models for each protein (false-discovery rate <0.1). Additional columns refer to the proportion of significant regression models in which the protein is increasing or decreasing; proteins with ten or more hits are listed. [file crc-25-0507_supplementary_table_s13_suppst13.docx]

**Supplementary Table S13.** Proteomic analysis of plasma, urine, and buffy coat revealed a distinct association between BPM31510IV treatment and the regulation of complement and coagulation events. Total hits are the number of significant regression models for each protein (false-discovery rate <0.1). Additional columns refer to the proportion of significant regression models in which the protein is increasing or decreasing; proteins with ten or more hits are listed.

| **Protein** | **Description** | **Total Hits** | **% hits going up** | **% hits going down** |
| --- | --- | --- | --- | --- |
| F2 | Prothrombin | 28 | 18 | 82 |
| PROS1 | Vitamin K-dependent protein S | 21 | 24 | 76 |
| C6 | Complement component C6 | 20 | 20 | 80 |
| F13A1 | Coagulation factor XIII A chain | 18 | 67 | 33 |
| SERPING1 | Plasma protease C1 inhibitor | 18 | 67 | 33 |
| C9 | Complement component C9 | 17 | 35 | 65 |
| CLU | Clusterin | 17 | 59 | 41 |
| FGA | Fibrinogen alpha chain | 17 | 24 | 76 |
| SERPINC1 | Antithrombin-III | 17 | 41 | 59 |
| F10 | Coagulation factor X | 16 | 25 | 75 |
| C7 | Complement component C7 | 15 | 67 | 33 |
| PROC | Vitamin K-dependent protein C | 15 | 33 | 67 |
| F11 | Coagulation factor XI | 14 | 57 | 43 |
| F9 | Coagulation factor IX | 13 | 31 | 69 |
| KLKB1 | Plasma kallikrein | 13 | 31 | 69 |
| C1S | Complement C1s subcomponent | 12 | 58 | 42 |
| F12 | Coagulation factor XII | 12 | 42 | 58 |
| FGB | Fibrinogen beta chain | 12 | 33 | 67 |
| KNG1 | Isoform LMW of Kininogen-1 | 12 | 25 | 75 |
| C8A | Complement component C8 alpha chain | 11 | 27 | 73 |
| C8G | Complement component C8 gamma chain | 11 | 36 | 64 |
| FGG | Fibrinogen gamma chain | 10 | 40 | 60 |
