# Supplementary material for: Phase Ia/b Multicenter Study of BPM31510IV Targeting Mitochondrial Metabolism/Warburg Effect as Monotherapy and Combination Chemotherapy in Solid Tumor Patients
Source: Cancer Res Commun. 2025 Dec 24;5(12):2207–23. doi: 10.1158/2767-9764.CRC-25-0507 (PMC12727275; doi:10.1158/2767-9764.CRC-25-0507)
Supplement: Supplementary Table S14 — Proteins detected in plasma, urine, or buffy coat that correlate with metabolic changes. Further information on the column names is provided in the Patients and Methods and the legend for Table S13. [file crc-25-0507_supplementary_table_s14_suppst14.docx]

**Supplementary Table S14.** Proteins detected in plasma, urine, or buffy coat that correlate with metabolic changes. Further information on the column names is provided in the Patients and Methods and the legend for Table S13.

| **Protein** | **Description** | **Total Hits** | **% hits going up** |
| --- | --- | --- | --- |
| ALDOA | Fructose-bisphosphate aldolase A | 14 | 43 |
| AGT | Angiotensinogen | 13 | 69 |
| CAT | Catalase | 11 | 64 |
| ALDOB | Fructose-bisphosphate aldolase B | 10 | 80 |
| ENO1 | Alpha-enolase | 10 | 80 |
| LBP | Lipopolysaccharide-binding protein | 10 | 60 |
| TALDO1 | Transaldolase | 10 | 80 |
| PFKP | 6-phosphofructokinase type C | 9 | 67 |
| HADHA | Trifunctional enzyme subunit alpha, mitochondrial | 8 | 62 |
| IDH1 | Isocitrate dehydrogenase (NADP), cytoplasmic | 8 | 38 |
| IDH2 | Isocitrate dehydrogenase (NADP), mitochondrial | 8 | 88 |
| MDH1 | Malate dehydrogenase, cytoplasmic | 8 | 75 |
| GLUD1 | Glutamate dehydrogenase 1, mitochondrial | 7 | 43 |
| GP1 | Glucose-6-phosphate isomerase | 7 | 86 |
| PGK1 | Phosphoglycerate kinase 1 | 7 | 57 |
| ECHS1 | Enoyl-CoA hydratase, mitochondrial | 6 | 83 |
| MDH2 | Malate dehydrogenase, mitochondrial | 6 | 83 |
| PRPS1 | Ribose-phosphate pyrophosphokinase 1 | 6 | 83 |
