# Supplementary material for: Phase Ia/b Multicenter Study of BPM31510IV Targeting Mitochondrial Metabolism/Warburg Effect as Monotherapy and Combination Chemotherapy in Solid Tumor Patients
Source: Cancer Res Commun. 2025 Dec 24;5(12):2207–23. doi: 10.1158/2767-9764.CRC-25-0507 (PMC12727275; doi:10.1158/2767-9764.CRC-25-0507)
Supplement: Supplementary Table S15 — Metabolites detected in plasma or urine that correlate with systemic protein changes. Further information on the column names is provided in the Patients and Methods and the legend for Table S13. [file crc-25-0507_supplementary_table_s15_suppst15.docx]

**Supplementary Table S15.** Metabolites detected in plasma or urine that correlate with systemic protein changes. Further information on the column names is provided in the Patients and Methods and the legend for Table S13.

| **Metabolite** | **Total Hits** | **% hits going up** |
| --- | --- | --- |
| Valine | 45 | 22 |
| Serine | 39 | 31 |
| Cysteine | 36 | 61 |
| Arginine | 35 | 20 |
| Leucine | 34 | 38 |
| Methionine | 33 | 33 |
| Glycine | 31 | 32 |
| Isoleucine | 30 | 37 |
| Lactic acid | 25 | 60 |
| Glutamic acid | 22 | 36 |
| Pyruvate | 22 | 68 |
| Tyrosine | 21 | 43 |
| Asparagine | 20 | 25 |
| Glutamine | 20 | 40 |
| Histidine | 20 | 25 |
| Malic acid | 18 | 39 |
| Aspartate | 15 | 20 |
| Alanine10 | 12 | 33 |
| Citric Acid | 10 | 90 |
| Fumarate | 10 | 60 |
| Fructose-6-phosphate | 9 | 67 |
| Glucose | 9 | 44 |
| Glucose-6-phosphate | 7 | 57 |
| Succinate | 7 | 71 |
