# Supplementary material for: Phase Ia/b Multicenter Study of BPM31510IV Targeting Mitochondrial Metabolism/Warburg Effect as Monotherapy and Combination Chemotherapy in Solid Tumor Patients
Source: Cancer Res Commun. 2025 Dec 24;5(12):2207–23. doi: 10.1158/2767-9764.CRC-25-0507 (PMC12727275; doi:10.1158/2767-9764.CRC-25-0507)
Supplement: Supplementary Table S16 — Changes in specific plasmalogen molecular species based on regression analysis. Further information on the column names is provided in the Patients and Methods and the legend for Table S13. [file crc-25-0507_supplementary_table_s16_suppst16.docx]

**Supplementary Table S16.** Changes in specific plasmalogen molecular species based on regression analysis. Further information on the column names is provided in the Patients and Methods and the legend for Table S13.

| **Lipid** | **Total Hits** | **% hits going up** |
| --- | --- | --- |
| PC-0-38:5 | 18 | 100 |
| PC-0-36:4 | 17 | 100 |
| PC-0-38:4 | 17 | 100 |
| PC-0-34:1 | 13 | 100 |
| PC-0-36:3 | 13 | 100 |
| PC-0-38:6 | 11 | 100 |
| PC-0-32:0 | 10 | 100 |
| PC-0-34:3 | 9 | 100 |
| PC-0-40:5 | 9 | 100 |
| PC-0-34:0 | 7 | 100 |
| PC-0-36:5 | 7 | 100 |
| PC-0-38:3 | 7 | 100 |
| PC-0-34:2 | 6 | 100 |
| PC-0-40:6 | 6 | 100 |
