# Supplementary material for: Phase Ia/b Multicenter Study of BPM31510IV Targeting Mitochondrial Metabolism/Warburg Effect as Monotherapy and Combination Chemotherapy in Solid Tumor Patients
Source: Cancer Res Commun. 2025 Dec 24;5(12):2207–23. doi: 10.1158/2767-9764.CRC-25-0507 (PMC12727275; doi:10.1158/2767-9764.CRC-25-0507)
Supplement: Supplementary Table S17 — Plasma metabolites positively associated with coenzyme Q10 (CoQ10) levels. [file crc-25-0507_supplementary_table_s17_suppst17.docx]

**Supplementary Table S17.** Plasma metabolites positively associated with coenzyme Q10 (CoQ_10_) levels.

| **Name** | **Slope** | **SE** | ***p*-value** | ***q*-value** |
| --- | --- | --- | --- | --- |
| GLYCEROPHOSPHOCHOLINE | 9.03E-04 | 1.38E-04 | 9.27E-11 | 1.89E-08 |
| 5-METHYL-TETRAHYDROFOLATE | 7.97E-04 | 2.34E-04 | 6.73E-04 | 7.63E-03 |
| 3-METHYLHISTIDINE | 7.73E-04 | 2.73E-04 | 4.78E-03 | 2.12E-02 |
| 2-AMINOBUTYRATE | 6.59E-04 | 1.10E-04 | 3.35E-09 | 3.41E-07 |
| MYRISTOYLCARNITINE | 6.13E-04 | 1.57E-04 | 1.04E-04 | 3.52E-03 |
| ADENOSINE | 5.86E-04 | 1.75E-04 | 8.48E-04 | 8.19E-03 |
| SPERMIDINE | 5.85E-04 | 1.60E-04 | 2.76E-04 | 4.33E-03 |
| CARBAMOYL PHOSPHATE | 5.51E-04 | 1.60E-04 | 6.14E-04 | 7.37E-03 |
| PHOSPHORYLCHOLINE | 5.07E-04 | 9.73E-05 | 2.33E-07 | 1.58E-05 |
| TETRADECENOYLCARNITINE | 4.61E-04 | 1.39E-04 | 9.70E-04 | 8.19E-03 |
| TETRADECADIENCARNITINE | 4.59E-04 | 1.27E-04 | 3.18E-04 | 4.33E-03 |
| GUANINE MONOPHOSPHATE | 4.32E-04 | 1.76E-04 | 1.45E-02 | 4.61E-02 |
| TETRADECANEDIOIC ACID | 4.12E-04 | 1.26E-04 | 1.11E-03 | 8.19E-03 |
| 9-DECENOYLCARNITINE | 4.08E-04 | 1.37E-04 | 3.04E-03 | 1.51E-02 |
| DECANOYLCARNITINE | 3.90E-04 | 1.19E-04 | 1.12E-03 | 8.19E-03 |
| DECENOYLCARNITINE | 3.90E-04 | 1.19E-04 | 1.12E-03 | 8.19E-03 |
| OCTANOYLCARNITINE | 3.90E-04 | 1.19E-04 | 1.12E-03 | 8.19E-03 |
| EICOSATETRAENOIC ACID | 3.86E-04 | 1.40E-04 | 5.92E-03 | 2.51E-02 |
| OLEOYLCARNITINE | 3.86E-04 | 1.07E-04 | 3.14E-04 | 4.33E-03 |
| HEXADECANDIOIC ACID | 3.85E-04 | 1.10E-04 | 5.10E-04 | 6.49E-03 |
| PALMITOYLCARNITINE | 3.84E-04 | 1.05E-04 | 2.62E-04 | 4.33E-03 |
| CYTIDINE MONOPHOSPHATE | 3.82E-04 | 1.52E-04 | 1.20E-02 | 3.95E-02 |
| DODECANOYLCARNITINE | 3.68E-04 | 1.41E-04 | 9.27E-03 | 3.38E-02 |
| DODECENOYLCARNITINE | 3.68E-04 | 1.41E-04 | 9.27E-03 | 3.38E-02 |
| STEAROYLCARNITINE | 3.55E-04 | 1.34E-04 | 8.41E-03 | 3.24E-02 |
| THIAMINE-PHOSPHATE | 3.41E-04 | 1.02E-04 | 8.75E-04 | 8.19E-03 |
| XANTHINE | 3.39E-04 | 1.04E-04 | 1.11E-03 | 8.19E-03 |
| HOMOSERINE | 3.32E-04 | 1.11E-04 | 2.82E-03 | 1.48E-02 |
| S-METHYL-CYSTEINE | 3.10E-04 | 9.44E-05 | 1.05E-03 | 8.19E-03 |
| MYO-INOSITOL PHOSPHATE | 2.97E-04 | 1.16E-04 | 1.06E-02 | 3.66E-02 |
| HEXANOYLCARNITINE | 2.88E-04 | 9.99E-05 | 4.06E-03 | 1.93E-02 |
| FLAVIN ADENINE DINUCLEOTIDE | 2.85E-04 | 8.92E-05 | 1.43E-03 | 8.56E-03 |
| S-ADENOSYL-L-HOMOCYSTEINE | 2.53E-04 | 8.18E-05 | 2.03E-03 | 1.18E-02 |
| N-CARBAMOYL-L-ASPARTATE | 2.53E-04 | 9.28E-05 | 6.59E-03 | 2.63E-02 |
| SPHINGOSINE 1-PHOSPHATE | 2.22E-04 | 8.87E-05 | 1.26E-02 | 4.07E-02 |
| XANTHOSINE | 2.19E-04 | 7.68E-05 | 4.55E-03 | 2.06E-02 |
| METHIONINE | 2.00E-04 | 7.26E-05 | 6.06E-03 | 2.52E-02 |
| O-ACETYL-L-SERINE | 1.92E-04 | 6.28E-05 | 2.31E-03 | 1.31E-02 |
| ETHANOLAMINE | 1.73E-04 | 4.68E-05 | 2.33E-04 | 4.33E-03 |
| DEOXYINOSINE | 1.43E-04 | 5.52E-05 | 9.65E-03 | 3.42E-02 |
| Slope and standard error (SE) values for the linear relationship between metabolite level and CoQ_10_ levels in a mixed-effect linear model (see Methods). The slope values are in terms of a log2 unit of change in the level of the indicated metabolite per log2 unit change in CoQ_10_ level. Metabolites with a *q-*value <0.05 are listed. | | | | |
