# Supplementary material for: Phase Ia/b Multicenter Study of BPM31510IV Targeting Mitochondrial Metabolism/Warburg Effect as Monotherapy and Combination Chemotherapy in Solid Tumor Patients
Source: Cancer Res Commun. 2025 Dec 24;5(12):2207–23. doi: 10.1158/2767-9764.CRC-25-0507 (PMC12727275; doi:10.1158/2767-9764.CRC-25-0507)
Supplement: Supplementary Figure S1 — Kaplan–Meier curves showing median progression-free survival for patients in each dosing cohort and treatment arm. [file crc-25-0507_supplementary_figure_s1_suppsf1.docx]

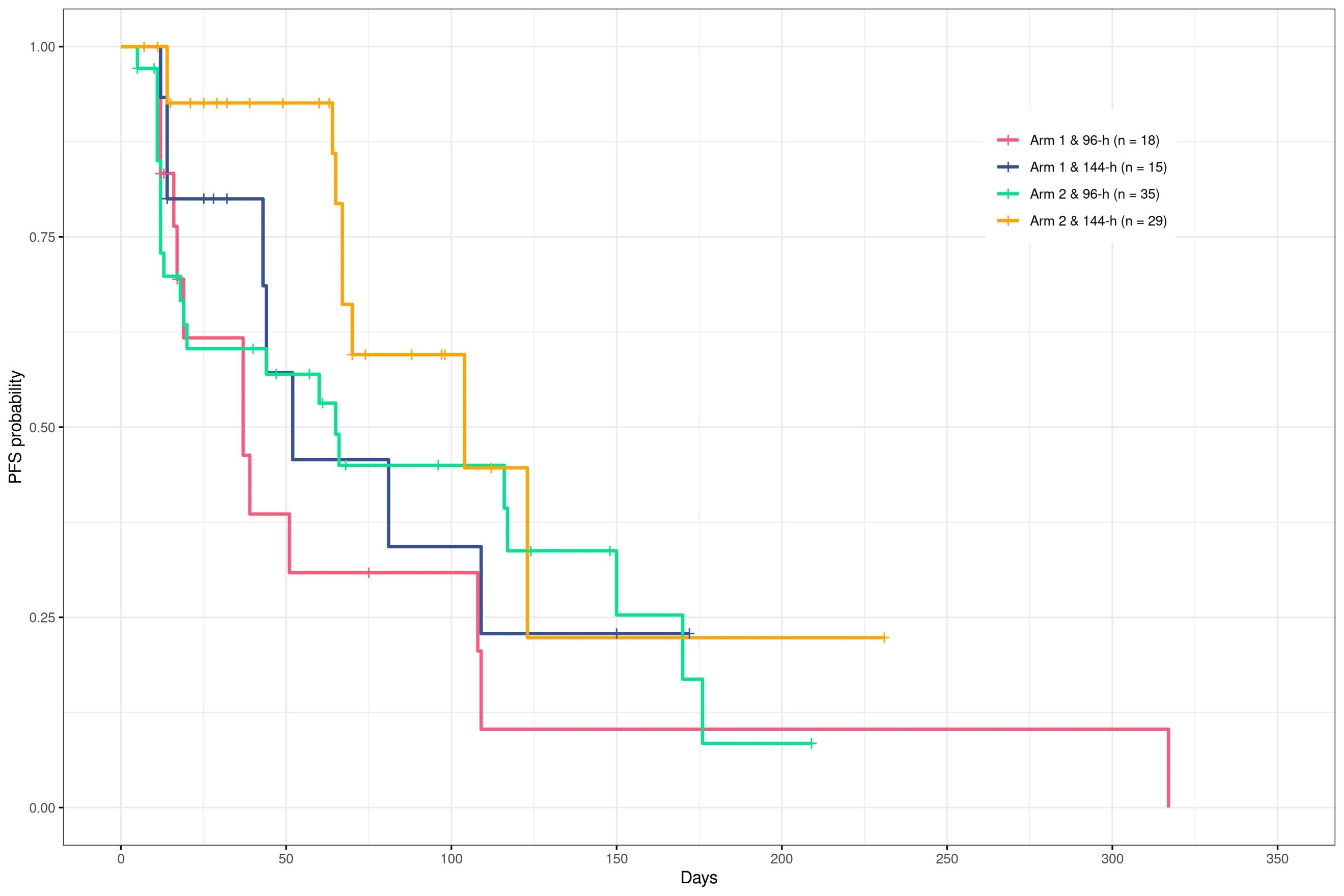


**Supplementary Figure S1.** Kaplan–Meier curves showing median progression-free survival for patients in each dosing cohort and treatment arm.
