# Supplementary material for: Phase Ia/b Multicenter Study of BPM31510IV Targeting Mitochondrial Metabolism/Warburg Effect as Monotherapy and Combination Chemotherapy in Solid Tumor Patients
Source: Cancer Res Commun. 2025 Dec 24;5(12):2207–23. doi: 10.1158/2767-9764.CRC-25-0507 (PMC12727275; doi:10.1158/2767-9764.CRC-25-0507)

**Supplementary Figure S2.** Mean plasma concentrations of BPM31510IV at Cycle 1 Week 1. Graphs show mean BPM31510IV concentrations for patients receiving 96-h infusion in Arm 1 (A) and Arm 2 (B) and patients receiving 144-h infusion in Arm 1 (C) and Arm 2 (D).

171 mg/kg

215 mg/kg

137 mg/kg

110 mg/kg

215 mg/kg

171 mg/kg

137 mg/kg

88 mg/kg

66 mg/kg

50 mg/kg

110 mg/kg

66 mg/kg

88 mg/kg


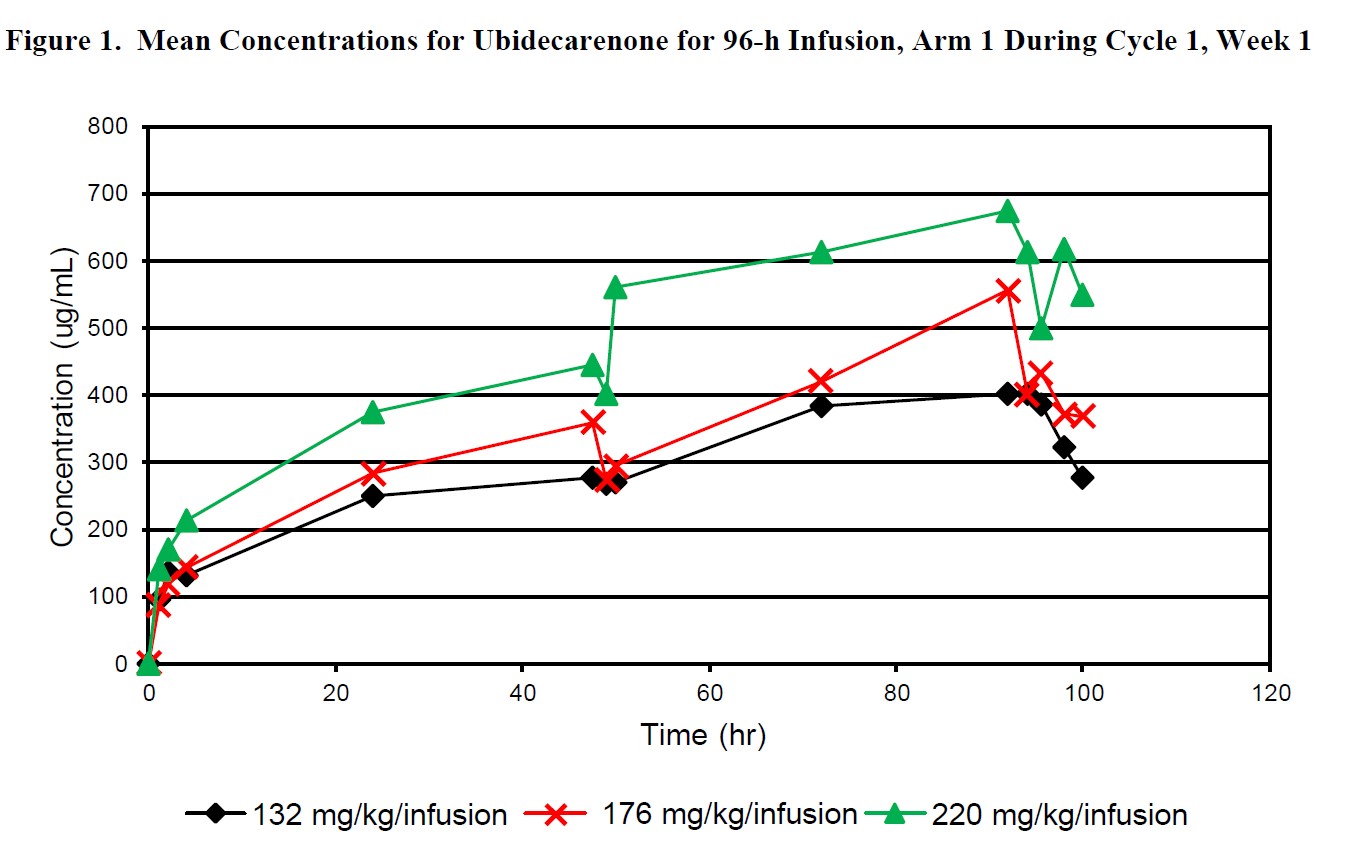

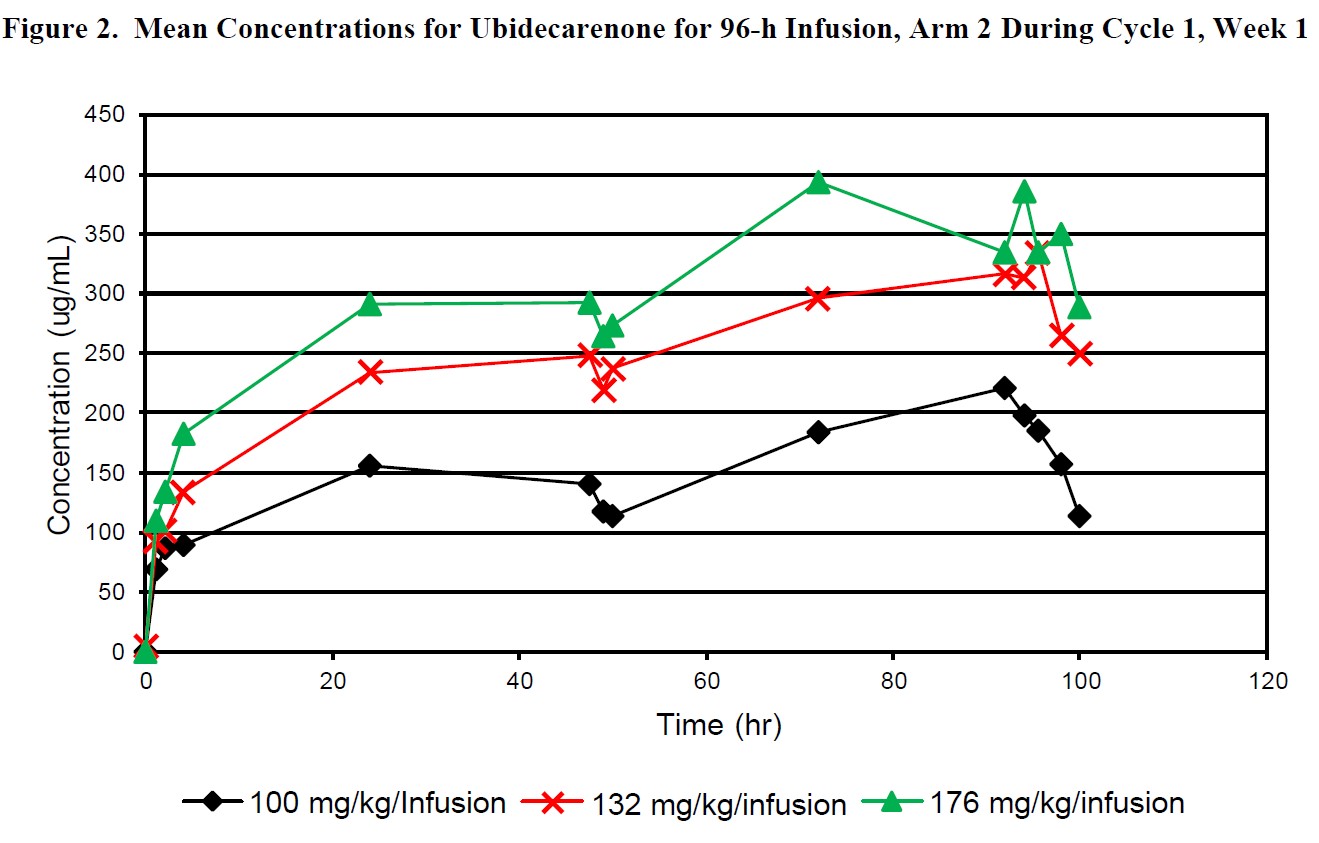


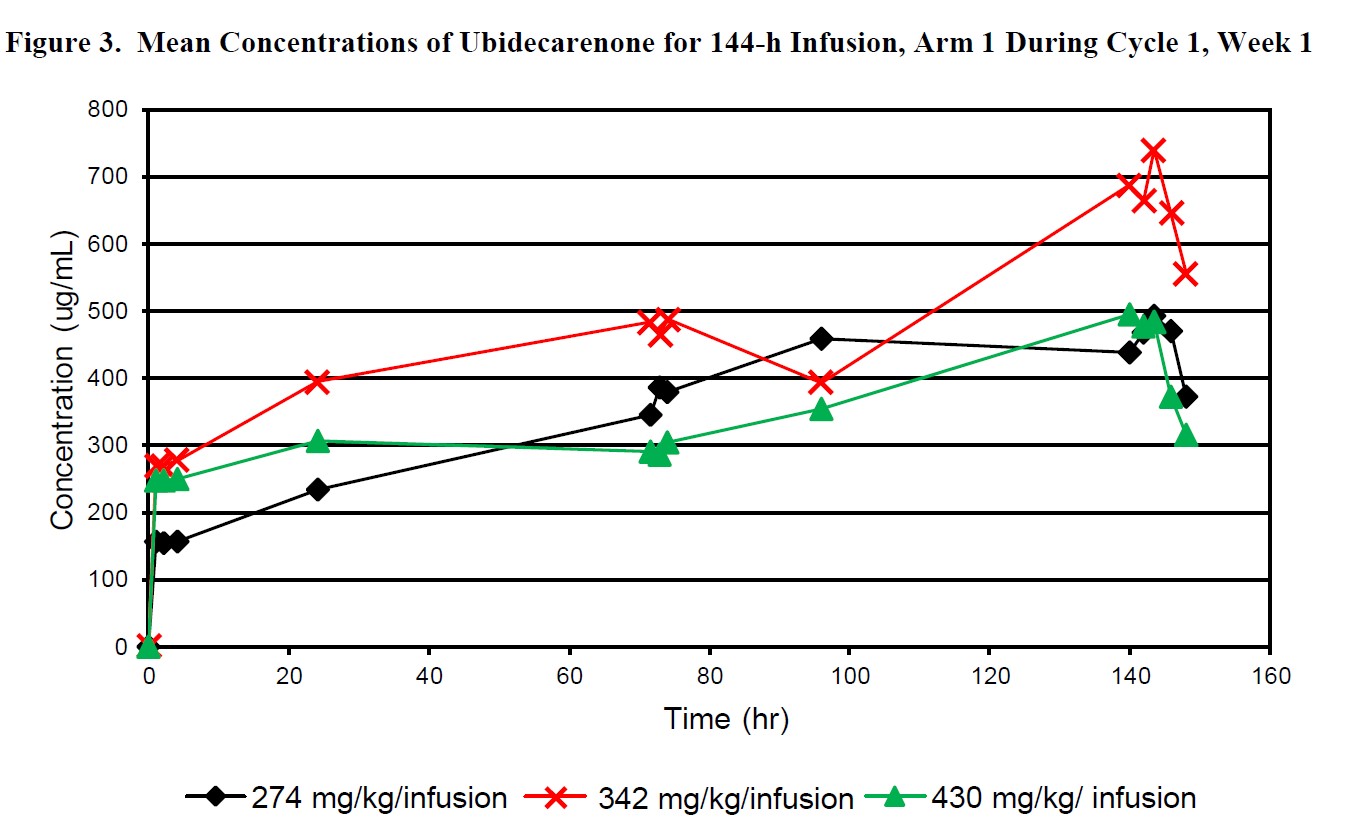

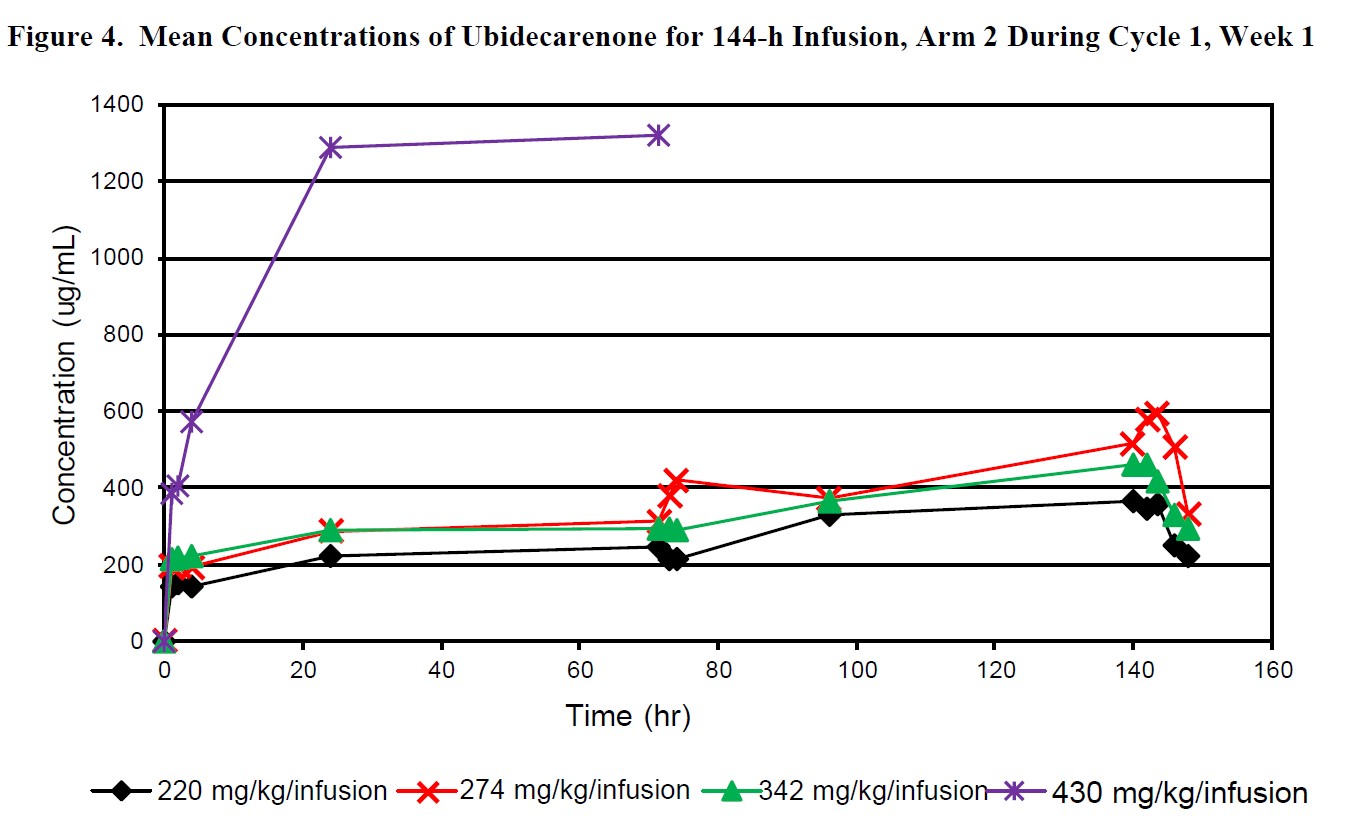

Supplement: Supplementary Figure S2 — Mean plasma concentrations of BPM31510IV at Cycle 1 Week 1. Graphs show mean BPM31510IV concentrations for patients receiving 96-h infusion in Arm 1 (A) and Arm 2 (B) and patients receiving 144-h infusion in Arm 1 (C) and Arm 2 (D). [file crc-25-0507_supplementary_figure_s2_suppsf2.docx]
