# Supplementary material for: Phase Ia/b Multicenter Study of BPM31510IV Targeting Mitochondrial Metabolism/Warburg Effect as Monotherapy and Combination Chemotherapy in Solid Tumor Patients
Source: Cancer Res Commun. 2025 Dec 24;5(12):2207–23. doi: 10.1158/2767-9764.CRC-25-0507 (PMC12727275; doi:10.1158/2767-9764.CRC-25-0507)
Supplement: Supplementary Figure S3 — Waterfall plots showing best response to BPM31510IV patients who had at least one tumor imaging assessment at or following Cycle 2, by cohort. A., Arm 1. B, Arm 2. [file crc-25-0507_supplementary_figure_s3_suppsf3.docx]

**
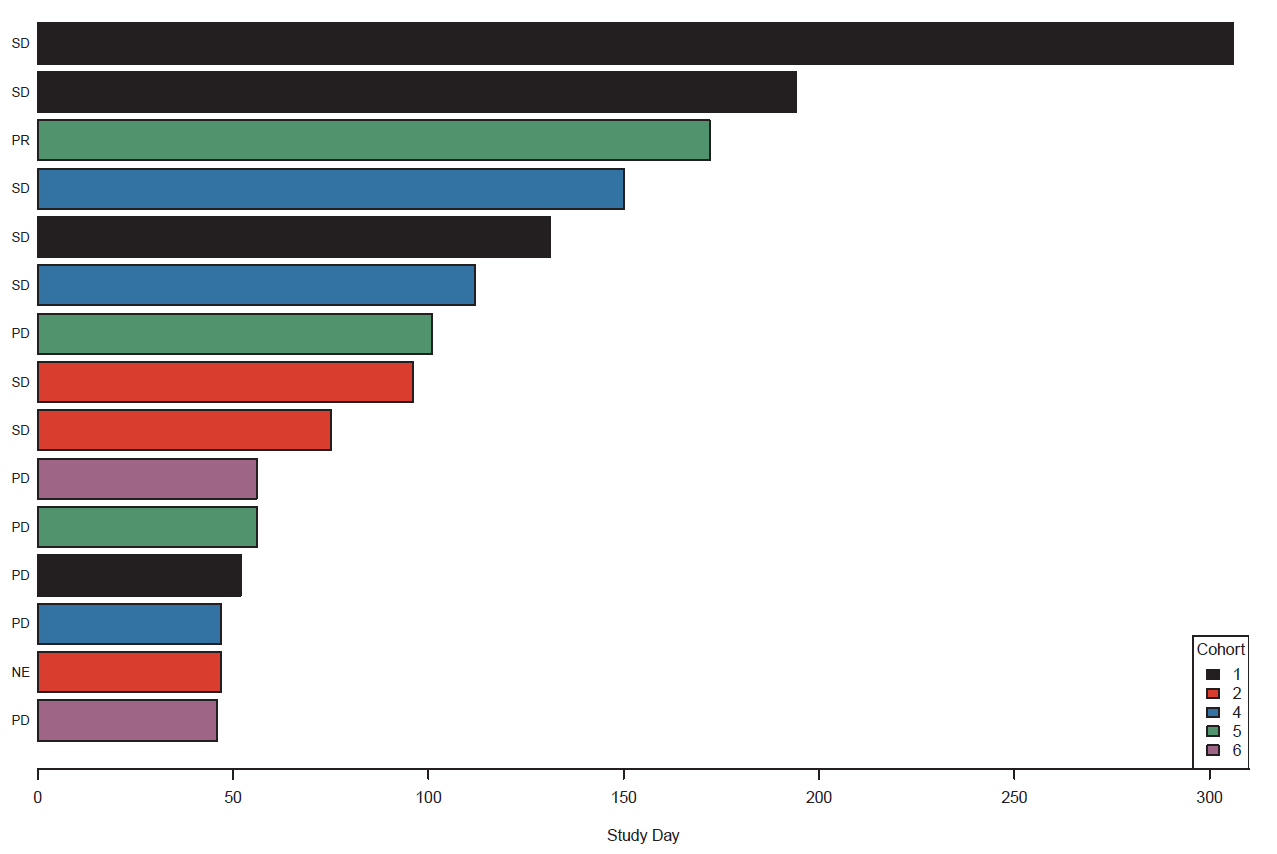
A.**


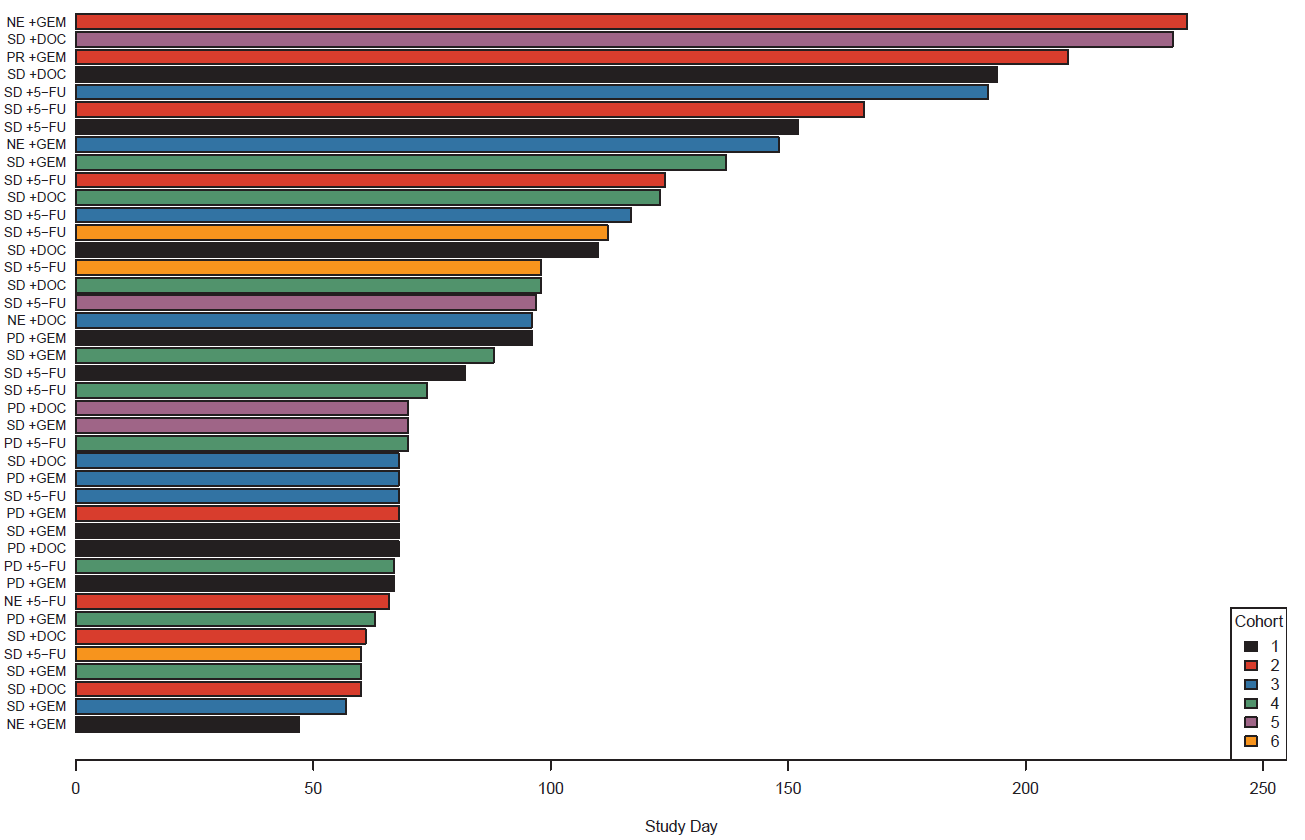
**B.**

**Supplementary Figure S3.** Waterfall plots showing best response to BPM31510IV patients who had at least one tumor imaging assessment at or following Cycle 2, by cohort. A., Arm 1. B, Arm 2. CR, complete response; PR, partial response; SD, stable disease; PD, progressive disease; NE, non-evaluable. 5-FU, 5-fluorouracil; Gem, Gemcitabine; Doc, Docetaxel. Cohorts ‒1 through 3 received two consecutive 48-h infusions of BPM31510IV, and cohorts 4 through 10 received two consecutive 72-h infusions of BPM31510IV.
