# Supplementary material for: Phase Ia/b Multicenter Study of BPM31510IV Targeting Mitochondrial Metabolism/Warburg Effect as Monotherapy and Combination Chemotherapy in Solid Tumor Patients
Source: Cancer Res Commun. 2025 Dec 24;5(12):2207–23. doi: 10.1158/2767-9764.CRC-25-0507 (PMC12727275; doi:10.1158/2767-9764.CRC-25-0507)
Supplement: Supplementary Figure S4 — Waterfall plot showing different combinations of chemotherapy for ARM2 and different doses of BPM31510IV. [file crc-25-0507_supplementary_figure_s4_suppsf4.docx]

**Supplementary Figure S4.** Waterfall plot showing different combinations of chemotherapy for ARM2 and different doses of BPM31510IV. 5-FU, 5-fluorouracil
